# Beyond Design: Ergonomic Numerical Evaluation of a Biomimetic Breast Prosthesis for Daily Use

**DOI:** 10.3390/biomimetics11080554

**Published:** 2026-08-04

**Authors:** Francisco Josué Hernández Rangel, Martha Angélica Cano Figueroa, Jorge Corona Castuera, José Eduardo Monsiváis Rocha, María Cruz Del Rocío Terrones Gurrola, Pedro Cruz Alcantar

**Affiliations:** 1 Doctoral Program in Advanced Manufacturing, CIATEQ A.C., San Luis Potosí Unit, San Luis Potosí C.P. 78395, Mexico; josue.hernandez@uaslp.mx; 2 CIATEQ A.C., Manzana 5, Lote 1, Distrito de Educación, Salud, Ciencia, Tecnología e Innovación (DESCTI), San Agustín Tlaxiaca C.P. 42162, Mexico; 3 CIATEQ A.C., Av. Manantiales No. 20, Parque Industrial Bernardo Quintana, El Marqués C.P. 76246, Mexico; jcorona@ciateq.mx; 4 Unidad Académica Multidisciplinaria Región Altiplano, Universidad Autónoma de San Luis Potosí UASLP–UAMRA, Carretera Cedral km, 5+600, Ejido San José de las Trojes, Matehuala C.P. 78700, Mexico; jose.monsivais@uaslp.mx (J.E.M.R.); rocio.terrones@uaslp.mx (M.C.D.R.T.G.); pedro.cruz@uaslp.mx (P.C.A.)

**Keywords:** external breast prosthesis, biomimetic design, heat dissipation, finite element, ergonomic evaluation

## Abstract

External breast prostheses remain the primary noninvasive alternative after mastectomy; however, most commercial designs do not adequately reproduce natural breast biomechanics, heat dissipation, and ventilation behavior during daily activities. This study presents an integrated numerical framework for the ergonomic evaluation of a biomimetic external breast prosthesis under realistic use conditions. A multilayer prosthesis–torso assembly was generated through 3D digitization and modeled using nonlinear hyperelastic finite element formulations. The proposed design incorporated biomimetic lobular internal architecture and microsphere-based posterior ventilation configurations to improve load distribution and heat dissipation. Dynamic behavior was evaluated through modal, harmonic, and spectral analyses, while thermal and airflow simulations were used to assess interface temperature reduction and ventilation efficiency. The results showed physiologically acceptable dynamic displacements, without critical stress concentrations and natural frequencies outside dominant gait excitation ranges. Additionally, biomimetic ventilation configurations reduced contact temperatures by up to 6.58 °C compared with conventional commercial geometries. Overall, the proposed architecture demonstrated mechanical stability, improved thermal performance, ergonomic compatibility, and potential for personalized prosthesis design.

## 1. Introduction

Breast cancer remains a major global health concern due to its high incidence and associated mortality among women, particularly in developing countries. Despite advances in diagnosis, many cases continue to be detected at advanced stages and may require radical surgical interventions, such as partial or total mastectomy [1,2]. Although reconstructive surgery is available to some patients, many women choose external breast prostheses as a noninvasive, immediate, and reversible alternative for restoring body symmetry after breast surgery. Regardless of advances in prosthetic materials and external morphology, most commercially available external breast prostheses have not been evaluated using integrated mechanical, thermal, and dry-airflow analyses under idealized conditions. In addition, few published studies on external breast prostheses have integrated biomimetic internal architectures with multiphysics numerical analysis and patient-specific parametric customization [3,4,5].

Natural breast tissue undergoes complex, dynamic deformations during daily activities, such as walking, climbing stairs, or using public transportation, due to inertial, gravitational, and contact forces, and this behavior reflects its highly deformable and viscoelastic nature. A prosthesis that fails to replicate these characteristics could cause discomfort, instability, excessive movement relative to the chest wall, or non-physiological load transmission, potentially compromising wearer comfort and postural balance. Ventilation and the predicted temperature at the prosthesis–torso interface are also important performance considerations.

Heat accumulation and restricted airflow at the prosthesis–torso interface may contribute to perspiration, skin irritation, discomfort, and increased risk of prosthesis detachment during extended wear, particularly in warm environmental conditions. Some commercial prostheses incorporate textured surfaces or posterior protrusions intended to facilitate ventilation. However, their thermal and airflow performance is rarely quantified using coupled thermal or computational fluid dynamics analyses under representative daily-use conditions.

Biomimetic design strategies inspired by the lobular organization of mammary tissue may promote distributed load transfer and energy dissipation while producing deformation patterns closer to those of natural breast tissue. Such architecture may also enhance structural stability while preserving the soft and deformable mechanical behavior characteristic of native mammary tissue.

Despite recent advances in finite element modeling of breast biomechanics and external prosthesis design, most previous studies have focused on isolated aspects such as structural mechanics, external geometry, or material behavior. Limited research has addressed simultaneously integrating biomimetic internal architectures, dynamic biomechanical evaluation, thermal behavior, airflow-assisted ventilation analysis, and parametric patient-specific customization within a unified multiphysics framework.

Based on these considerations, it was hypothesized that incorporating biomimetic lobular internal architecture and posterior ventilation structures would promote distributed mechanical response, enhance heat dissipation, and more uniform load distribution compared with conventional external breast prosthesis designs.

Therefore, the objective of this study was to develop and computationally evaluate a biomimetic external breast prosthesis incorporating a parametric internal architecture and posterior ventilation structures under controlled idealized mechanical and environmental boundary conditions.

A parametric computer-aided design (CAD) framework with an integrated application programming interface (API) was developed to facilitate anthropometric geometric customization. The relationship implemented in the framework was informed by anthropometric measurements from 30 participants, while the specific geometry evaluated numerically corresponded to one anatomical medical mannequin configuration.

Evaluation criteria were established based on physiological soft tissue deformation ranges, vibratory perception thresholds, and heat dissipation limits.

This study presents an integrated computational framework for analyzing the biomechanical, thermal, and ergonomic behavior of biomimetic external breast prostheses under representative daily-use conditions. The obtained results represent numerical predictions based on idealized boundary conditions. The proposed bioinspired architecture was evaluated in terms of dynamic stability, mechanical compatibility, and thermal dissipation. The results should not be interpreted as validated evidence of human thermal comfort or clinical performance.

## 2. Materials and Methods

This work was conceived as a numerical, computational, biomimetic, parametric, and comparative investigation of an external breast prosthesis for women who had undergone a total mastectomy. The analysis evaluated the biomechanical, thermal, and ergonomic performance of the proposed design under controlled idealized loading and representative daily-use conditions through multiphysics numerical simulations, rather than constituting a clinical validation study.

**Figure 1:**
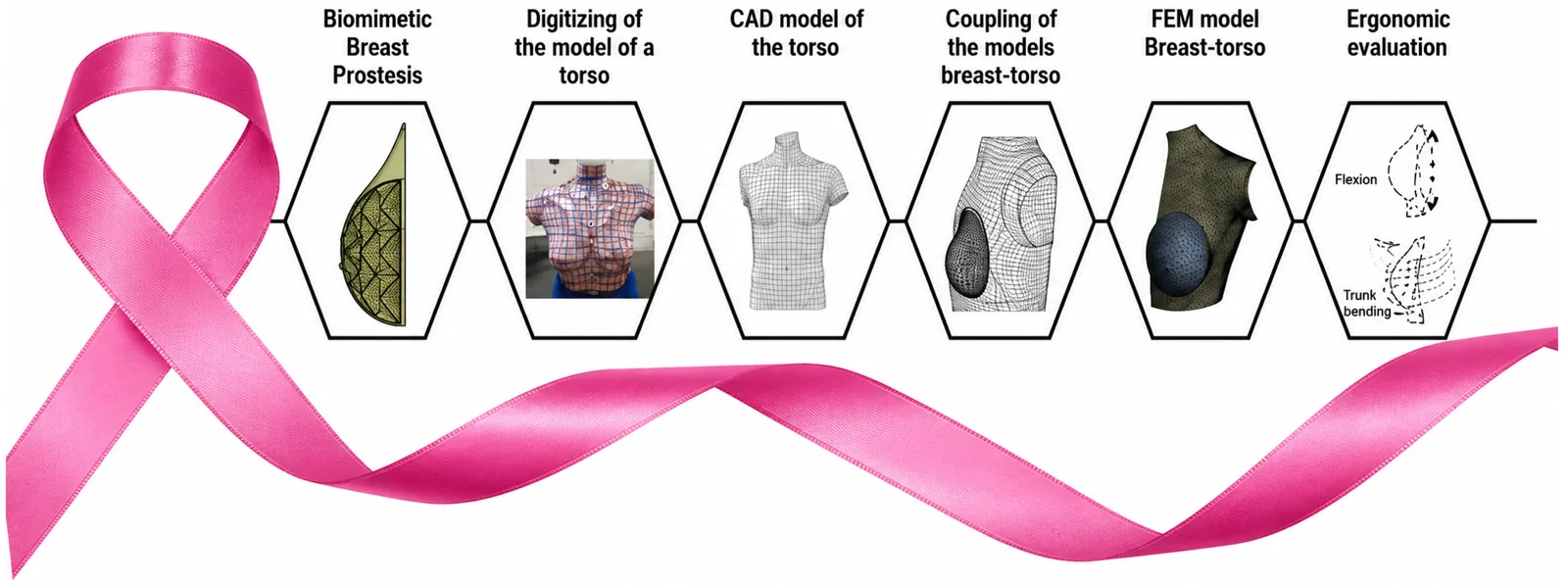
Integrated CAD-FEM methodology for personalized external breast prosthesis design.

The methodology for the ergonomic evaluation of the external breast prosthesis after total mastectomy consisted of six stages, as illustrated in Figure 1.

The proposed framework integrates all stages, from updating the prosthesis design to its subsequent ergonomic assessment. The details of each stage are described in the following sections.

### 2.1. Update of the Prosthesis Design Incorporating Anthropometric and Functional Improvements

The starting geometry was the external breast prosthesis model developed by Cruz et al. [6]. The model was dimensionally and morphologically updated using relationships identified from anthropometric measurements obtained from women who had undergone total mastectomy. The anthropometric dataset comprised 30 adult female volunteers aged 20–50 years who were recruited through non-probabilistic convenience sampling. The sample was used to examine relationships between breast and torso dimensions and to develop the equations implemented in the parametric computer-aided design macro. The dataset was intended for descriptive model development and was not used for population inference or statistical hypothesis testing. Therefore, no statistical power calculation was performed. Participant selection prioritized women between 20 and 50 years of age because of their greater likelihood of using external breast prostheses or undergoing breast reconstruction procedures [7]. The age distribution was as follows: 22 participants were 20–35 years old, five were 36–45 years old, and three were 46–50 years old. Age-related differences in torso geometry, breast morphology, tissue composition, and skin elasticity were not incorporated into the mannequin-based numerical model. Since the study involved only non-invasive external measurements and did not include medical interventions or the collection of sensitive clinical information, participants were informed about the study objectives and procedures and provided written informed consent before data collection. Inclusion criteria comprised previous unilateral total mastectomy, absence of active postoperative complications, and the ability to remain in a standing position during the measurement procedure. The exclusion criteria included severe thoracic deformities, active skin lesions, or recent surgical interventions that could alter torso geometry. Anthropometric data such as body morphology, breast contour, and thoracic dimensions were collected following the methodology proposed by Sohn and Kweon (2013) [8].

**Figure 2:**
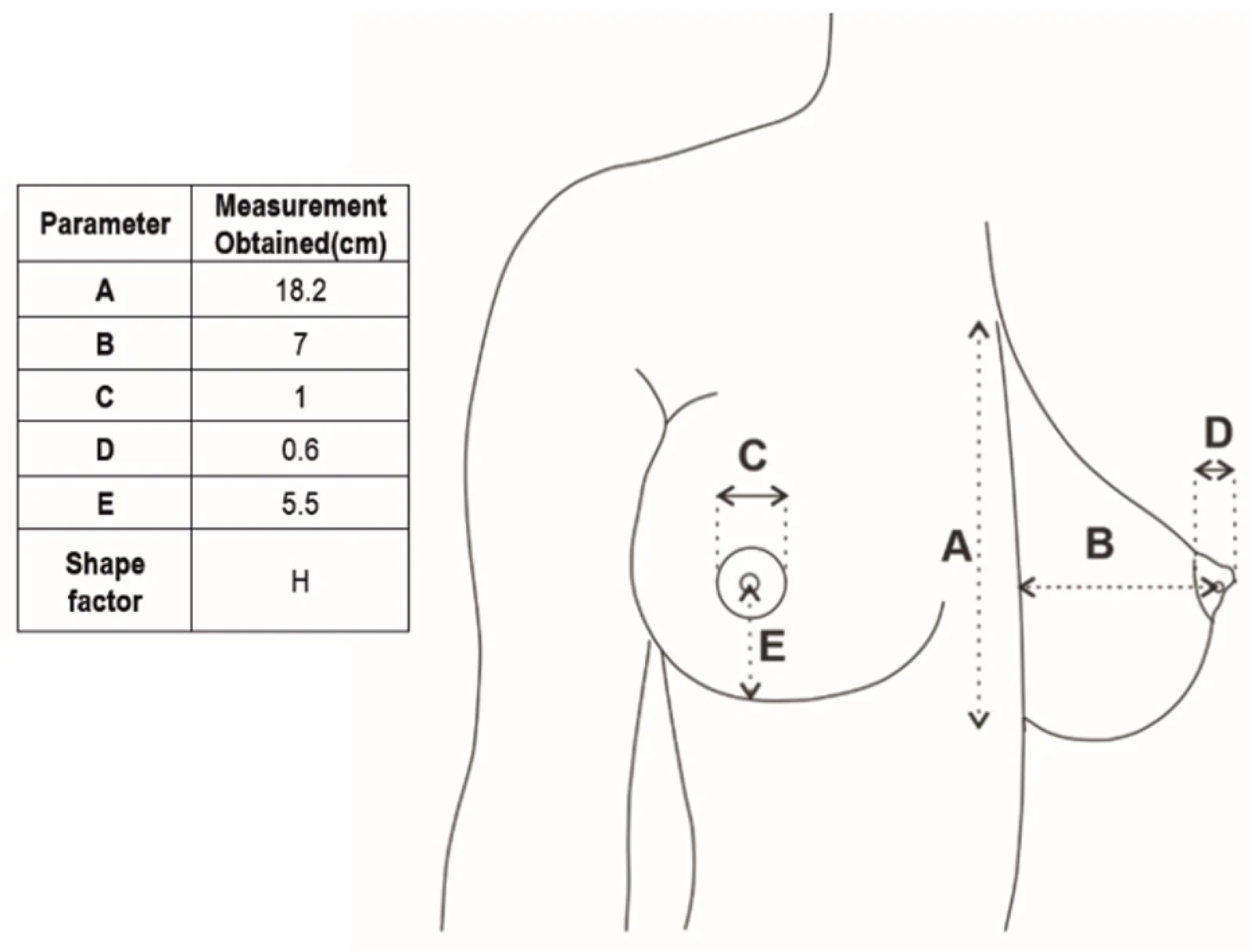
Anthropometric parameters and mean values used to update the prosthesis geometry in this work.

All participants provided written informed consent before data acquisition. The geometric parameters considered and the average values used to update the model are presented in Figure 2.

The anthropometric dataset was used to identify dimensional relationships and develop the equations implemented in the parametric CAD macro. The standard deviations, which were approximately ±10–20 mm for several linear dimensions, were consistent in accordance with the literature [9,10].

**Figure 3:**
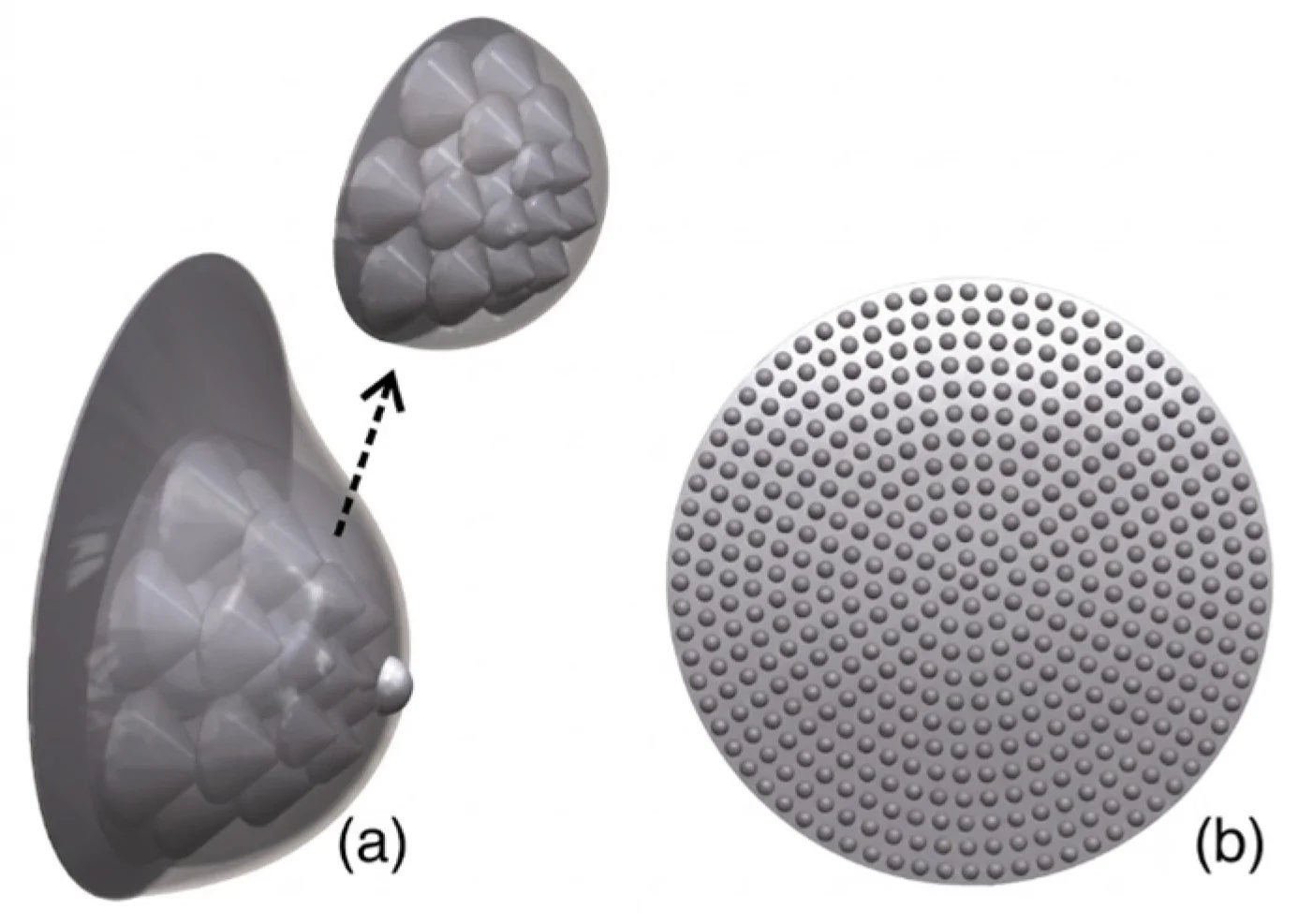
Updated prosthesis design: (**a**) internal triangular geometry inspired by the mammary lobular structure; (**b**) posterior surface with uniformly distributed spheres.

The updated prosthesis incorporated an internal triangular architecture inspired by the lobular arrangement of the mammary gland, as shown in Figure 3a. This geometry was introduced to investigate its predicted deformation and load-transfer behavior under the simulated conditions. Its inclusion should not be interpreted as evidence of mechanical superiority over a conventional homogeneous prosthesis because no direct mechanical comparison under identical conditions was performed in this investigation. The proposed geometry is aimed at reproducing more natural biomechanical behavior and simplifying the manufacturing process. Posterior microsphere patterns were incorporated to generate airflow pathways between the prosthesis and the torso surface, as shown in Figure 3b. Microsphere diameters ranging from 1 to 5 mm were evaluated under identical thermal and airflow boundary conditions. This comparison was limited to posterior airflow and heat-transfer behavior and did not constitute a mechanical comparison between internal prosthesis architectures.

### 2.2. Three-Dimensional Scanning of the Torso of a Medical Model and Coupling Between Prosthesis and Torso Models

**Figure 4:**
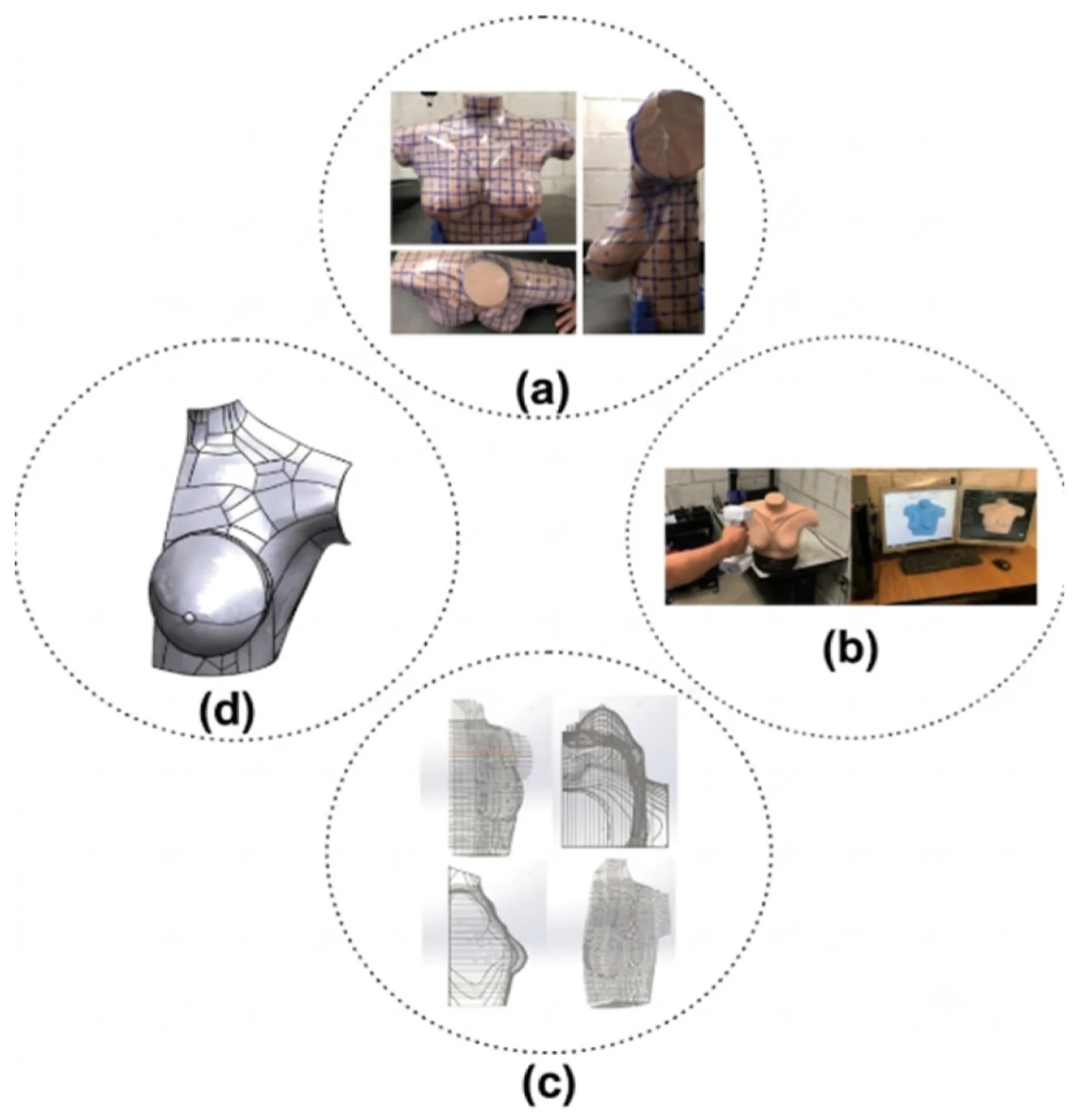
Torso digitization and prosthesis–torso coupling process: (**a**) coordinate acquisition using CMM; (**b**) 3D scanning and validation; (**c**) point cloud processing and surface reconstruction; (**d**) final prosthesis–torso assembly for numerical evaluation.

The torso was digitized using a combined methodology that integrated a coordinate measuring machine (CMM, Mitutoyo M574 Crysta-Plus located in Matehuala, San Luis Potosí, México), a 3D scanner (EinScan H located in the same place), and SolidWorks software 2024 to obtain an accurate and reproducible geometric model, as shown in Figure 4.

To minimize biological variability, a medical mannequin was used as a reference model to ensure controlled geometric conditions during digitization to improve reproducibility during the acquisition and reconstruction processes [11]. Although this approach reduces intersubject variability, it does not fully reproduce the deformability, anatomical asymmetries, or clinical variability of the human thorax, which are recognized as inherent limitations of the present numerical model. Therefore, the resulting geometry was not conceived as a definitive patient-specific model, but rather as a parametric baseline that can subsequently be customized through the developed CAD–API framework according to individual anthropometric characteristics.

The point cloud obtained through the CMM was generated from cutting lines separated by 1 cm in both the vertical and horizontal directions.

The model obtained by 3D scanning was used to verify and adjust the CMM coordinates. The procedure was repeated in three independent runs to assess repeatability, and the consolidated point cloud was processed in SolidWorks to generate the final geometric model. From this model, the breast region was isolated to simulate the condition after a total mastectomy, resulting in an average geometry used as a universal model for the numerical evaluation of the prosthesis-torso system.

**Figure 5:**
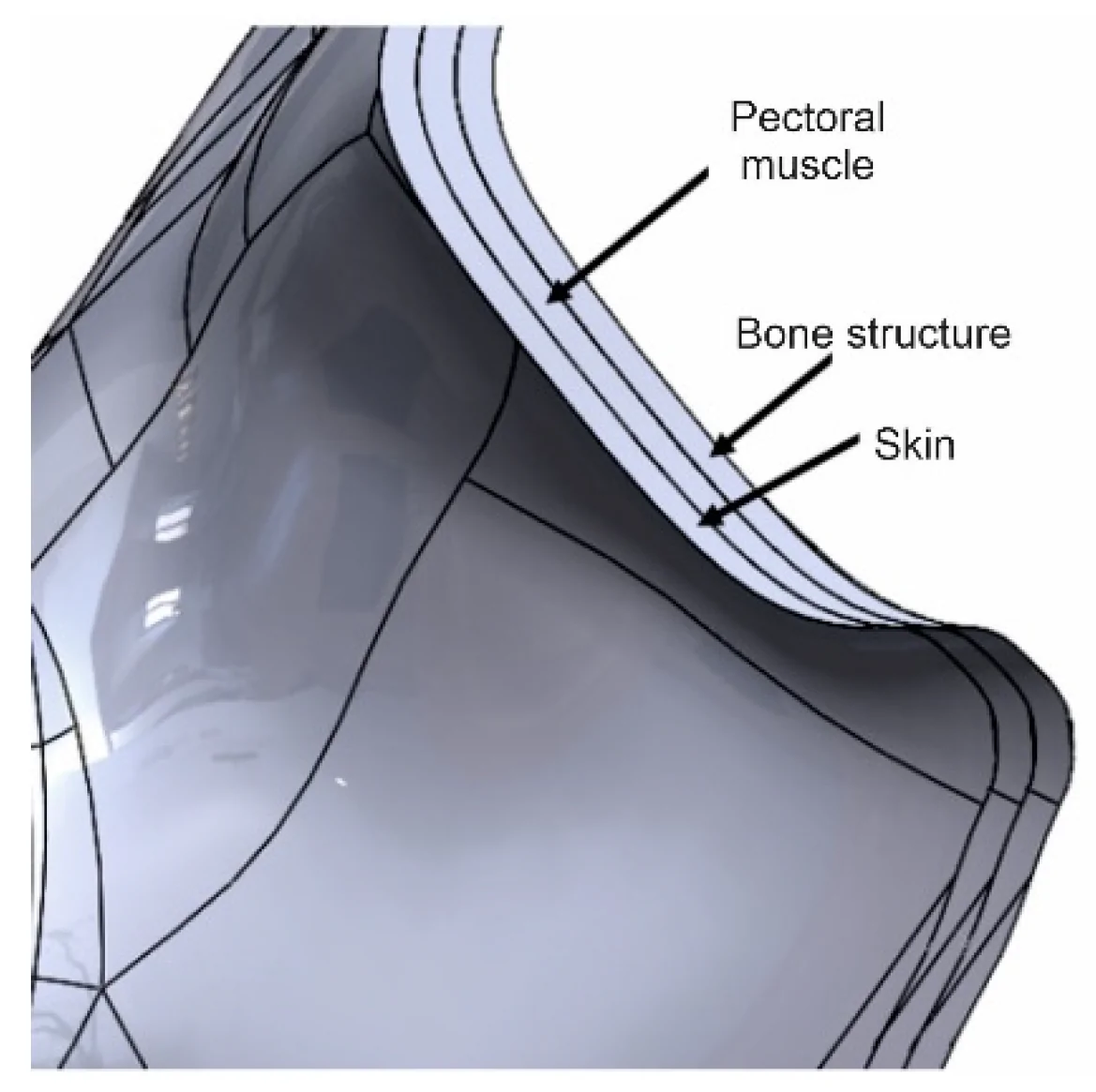
Multilayer torso model incorporating skin, muscle, and bone structures for ergonomic and dynamic evaluation of the prosthesis.

According to the methodology proposed by Mira et al. [12], three layers were incorporated into the torso model to represent the properties and behavior of the skin, muscle, and bone structure during the ergonomic and dynamic evaluation of the prosthesis. These layers are shown in Figure 5.

### 2.3. Construction of the FEM Model of the Prosthesis–Torso Assembly

**Figure 6:**
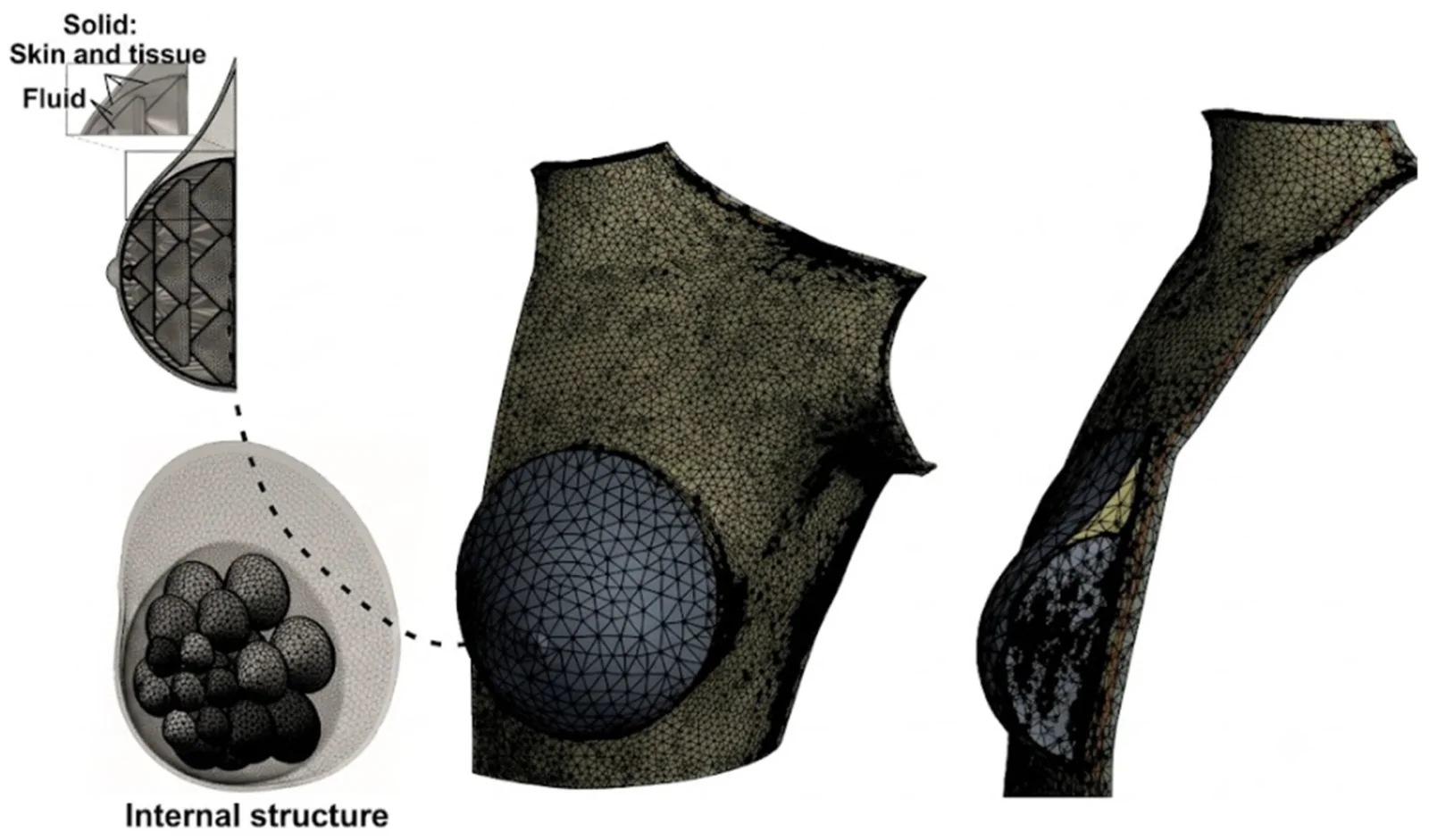
Finite element model of the prosthesis–torso assembly.

The mechanical and dynamic behavior of the prosthesis–torso assembly was evaluated using FEM as shown in Figure 6. The analyses were designed to compare predicted displacement, stress, strain, contact behavior, natural frequencies, and harmonic response under controlled idealized loading conditions.

Material domains were assigned constitutive properties obtained from previously published experimental data and finite element formulations. Skin and glandular tissue were represented using Neo-Hookean hyperelastic models. Adipose tissue was represented using a five-term Mooney–Rivlin model. The materials were assumed to be homogeneous and isotropic within each modeled domain. For the skin domain, the Neo-Hookean coefficients were C_10_ = 83.89 kPa and D_1_ = 0.00024 kPa. For glandular tissue, the corresponding coefficients were C_10_ = 1.678 kPa and D_1_ = 0.012 kPa. Adipose tissue was assigned a five-term Mooney–Rivlin model with C_10_ = 0.31, C_01_ = 0.30, C_20_ = 3.80, C_11_ = 2.25, and C_02_ = 4.72 kPa. These coefficients were adopted from Chen et al. [12].

Adipose and fibroglandular tissue, and the equivalent skin, were characterized by Young’s moduli of 2.5 kPa, 60 kPa, and 10 kPa (skin thickness of 2 mm), respectively. In the torso, bone tissue was defined with a modulus of 1.6 GPa and a density of 1850 kg/m^3^, while muscle tissue had a modulus of 30 kPa and a density of 1200 kg/m^3^. Poisson’s ratio of 0.49 was used for soft tissues [13].

The prosthesis was modeled in three distinct regions: external shell, internal structure, and internal spaces. The 2 mm-thick external shell was assigned to properties equivalent to human skin (E = 10 kPa). The internal structure was modeled as a Neo-Hookean material (E = 15 kPa), while the internal spaces were assigned properties equivalent to adipose tissue.

Meshing employed SOLID187 elements for regions with complex geometry and SOLID186 elements for torso regions with lower deformations and optimized via a convergence study with a variation criterion below 5% in maximum displacement. The final model consisted of 373,303 nodes and 177,269 elements. The finite element model of the prosthesis–torso assembly is shown below.

A nonlinear mechanical behavior with large deformations was assumed, which is appropriate for simulating soft biological tissues [13]. Contact between the prosthesis and torso was defined using a “no separation” formulation, accounting for the adhesive effect and mechanical support provided by clothing. This approach ensured load transfer while preventing solid interpenetration using Lagrange multipliers.

Contact between biological tissues (bone–muscle and skin–muscle interfaces) was modeled as frictional, using a friction coefficient of μ = 0.4, a value commonly adopted in biomechanical simulations of soft tissues [14,15].

Boundary conditions were defined to reproduce representative daily-use conditions of the prosthesis–torso system. The posterior and lateral regions of the torso were constrained to prevent rigid body motion while allowing local deformations in the prosthesis contact area. Gravitational acceleration of 9.81 m/s^2^ was applied in all mechanical and dynamic analyses to account for the self-weight of the prosthesis and torso tissues.

A modal analysis was performed over the 3–9 Hz interval to identify the natural frequencies and associated mode shapes of the prosthesis–torso assembly. The modal deformation contours represented normalized relative deformation patterns and not physical displacement amplitudes. Eight natural frequencies were identified within the evaluated interval. The first four frequencies were compared with values reported by Chen et al. [16] as a literature-based numerical reference. This comparison was not considered an experimental validation of the present model. The harmonic analysis was conducted over the same frequency interval using a sinusoidal force with an amplitude of 5 N representing typical manual contact forces. The force was applied normally to the upper external torso region as a controlled mechanical excitation. The prosthesis–torso interface was represented using idealized continuous contact associated with adhesive and garment support effects, rather than by applying external pressure constraints.

### 2.4. Ergonomic Evaluation Under Controlled Temperature, Ventilation, and Dynamic Loading Conditions, Defined According to Comfort and Performance Criteria

**Figure 7:**
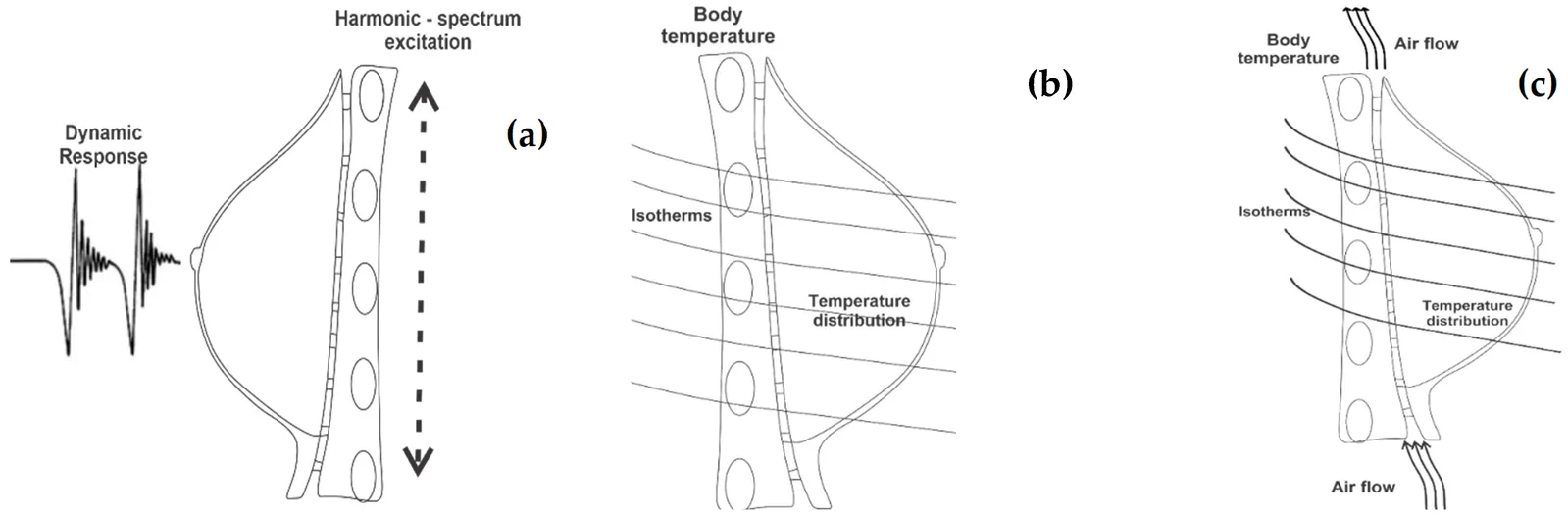
Ergonomic evaluation of the prosthesis–torso system: (**a**) dynamic analysis, (**b**) thermal analysis, and (**c**) ventilation analysis under controlled conditions.

The ergonomic evaluation was conducted through dynamic (Figure 7a), thermal (Figure 7b), and ventilation (Figure 7c) numerical analyses.

The dynamic analysis included a modal study and a harmonic-spectral analysis to characterize the vibrational response of the prosthesis–torso assembly. Natural frequencies, dominant shapes, and dynamic amplitudes were determined to identify frequency ranges or displacement levels that could be potentially critical from the standpoint of user comfort and safety.

Viscous material behavior and structural damping were not included in the harmonic model. Consequently, the predicted response amplitudes, particularly near natural frequencies, may be overestimated. The harmonic amplitudes were therefore interpreted as comparative numerical predictions and not as experimentally validated displacement values occurring during actual prosthesis use.

The thermal analysis evaluated the predicted temperature distribution and sensible heat transfer at the prosthesis–torso interface. The torso–interface temperature was set to 36.00 °C [17], while the inlet air temperature was defined as 24.5 °C. Thermophysical properties, including thermal conductivity, density, and specific heat capacity, were assigned to the biological and prosthetic domains using literature-derived values [18,19].

Heat transfer between the prosthesis and torso was modeled considering conductive contact and convective cooling induced by airflow at the interface. A convective boundary condition representative of indoor comfort environments was applied to the external surfaces exposed to air. A convective heat-transfer coefficient of 5 W/m^2^·K was applied to the surfaces exposed to ambient air. The thermal effect of clothing and adhesive fixation was indirectly represented through the defined prosthesis–torso contact conditions, although detailed thermal contact resistance and moisture transport phenomena were not explicitly modeled.

The resulting thermal gradient and steady-state temperature distribution were evaluated considering the ventilation effect induced by the supporting microspheres. The airflow analysis evaluated the velocity and temperature fields within the posterior space between the prosthesis and torso. All posterior configurations were analyzed using identical airflow and thermal boundary conditions. The air inlet temperature was set to 24.5 °C, the inlet velocity was 0.25 m/s, and the outlet was defined using atmospheric pressure, in accordance with the indoor comfort parameters established by NOM-001-STPS-2008 [19]. A low-intensity k–ε turbulence model was employed to represent airflow distribution and heat transfer at the prosthesis–torso interface more realistically, accounting for the combined effects of ambient airflow and body motion. For the CFD model, dry airflow was considered and did not include sweat, surface wetting, or evaporation in the model.

### 2.5. Ergonomic Evaluation Criteria

From a dynamic standpoint, it was considered acceptable for the natural frequencies of the prosthesis–torso system to lie outside the dominant range of physiological excitation associated with gait and daily activities (≈1–3 Hz) [20]. Dynamic amplitudes were evaluated using two reference levels: values ≤ 1 mm, associated with micro-movements generally imperceptible to the user, and maximum amplitudes ≤ 5 mm, which, although exceeding the fine perception threshold, remain within the range of normal physiological displacements of soft tissues during daily activities and are therefore considered biomechanically compatible and ergonomically tolerable [19,20].

From a mechanical perspective, acceptable behavior was defined as a global response comparable to the natural motion of breast tissue, with no localized stress concentrations or structural deformations that could compromise the integrity of the model, with stress remaining well below the elastic limit of the materials used.

Finally, in the thermal and ventilation analyses, ergonomic criteria were defined as a contact temperature below 33–34 °C, along with a minimum thermal reduction on the order of 3–5 °C at the prosthesis–torso interface compared to non-ventilated surfaces.

## 3. Results

The selected modal analysis range (3–9 Hz) was intentionally wider than the dominant excitation frequencies associated with human gait, which are generally reported to be between 1 and 3 Hz. This approach allowed for the evaluation of the dynamic response of the prosthesis–torso system both during normal locomotion and to higher-frequency excitations that may arise during running, jumping, or vibrations related to transportation [21,22,23,24]. This range enables the identification of the natural frequencies and vibration modes of the system. Eight vibration modes were identified within the analyzed frequency range. Among them, Modes 1, 2, and 6 were selected for detailed discussion because they exhibited the most representative global deformation patterns and stress distributions associated with realistic prosthesis motion during daily activities, whereas the remaining modes showed localized or less mechanically significant responses.

**Figure 8:**
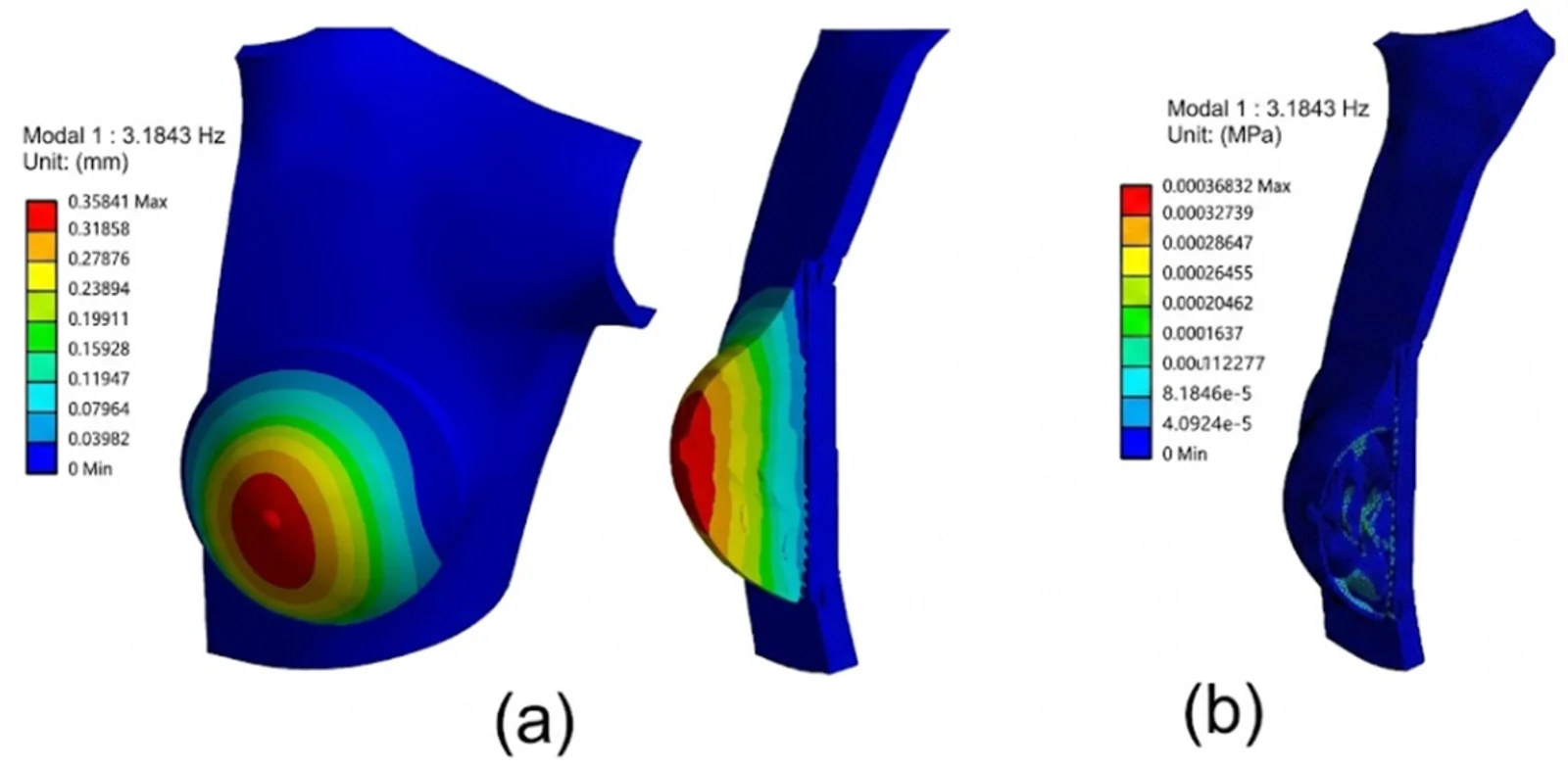
First vibration mode of the prosthesis–torso assembly (3.18 Hz). (**a**) Total displacement (max: 0.358 mm). (**b**) Equivalent stress distribution.

The first mode (Figure 8a) corresponds to a global prosthesis displacement associated with gravitational effects, exhibiting a natural frequency of 3.18 Hz and a maximum amplitude of 0.3584 mm. This mode does not compromise structural integrity nor induce significant alterations in the torso. The spherical geometry and lobular fit contributed to homogeneous stress distribution within the prosthesis (Figure 8b).

**Figure 9:**
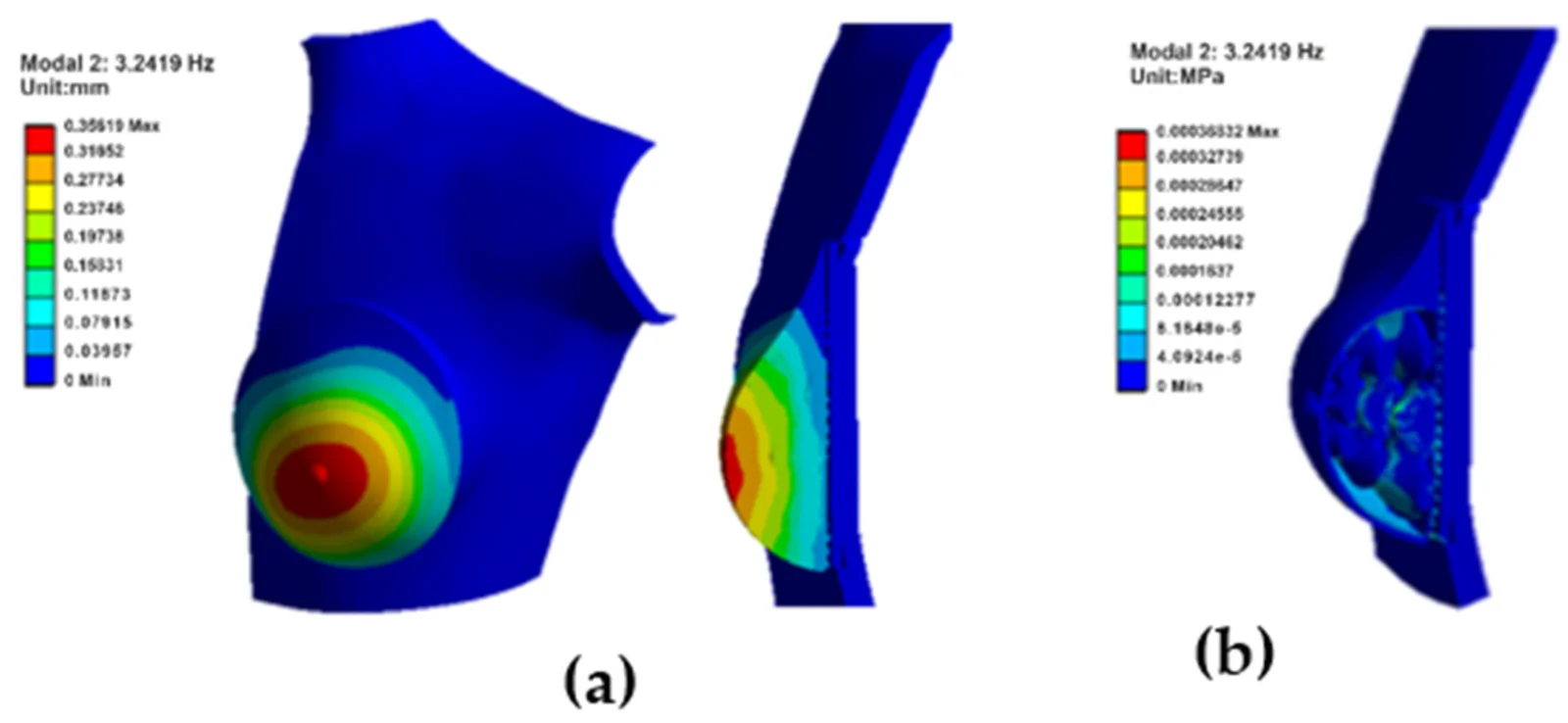
Second vibration mode of the prosthesis–torso assembly (3.24 Hz). (**a**) Total displacement (max: 0.356 mm). (**b**) Equivalent stress distribution.

The second vibration mode (Figure 9a) corresponds to an axial left-to-right motion at a frequency of 3.24 Hz with a maximum amplitude of 0.3561 mm. The internal stresses and deformations at the contact interface with the torso skin were minimal; therefore, this frequency was considered within the safe operating range of the prosthesis.

**Figure 10:**
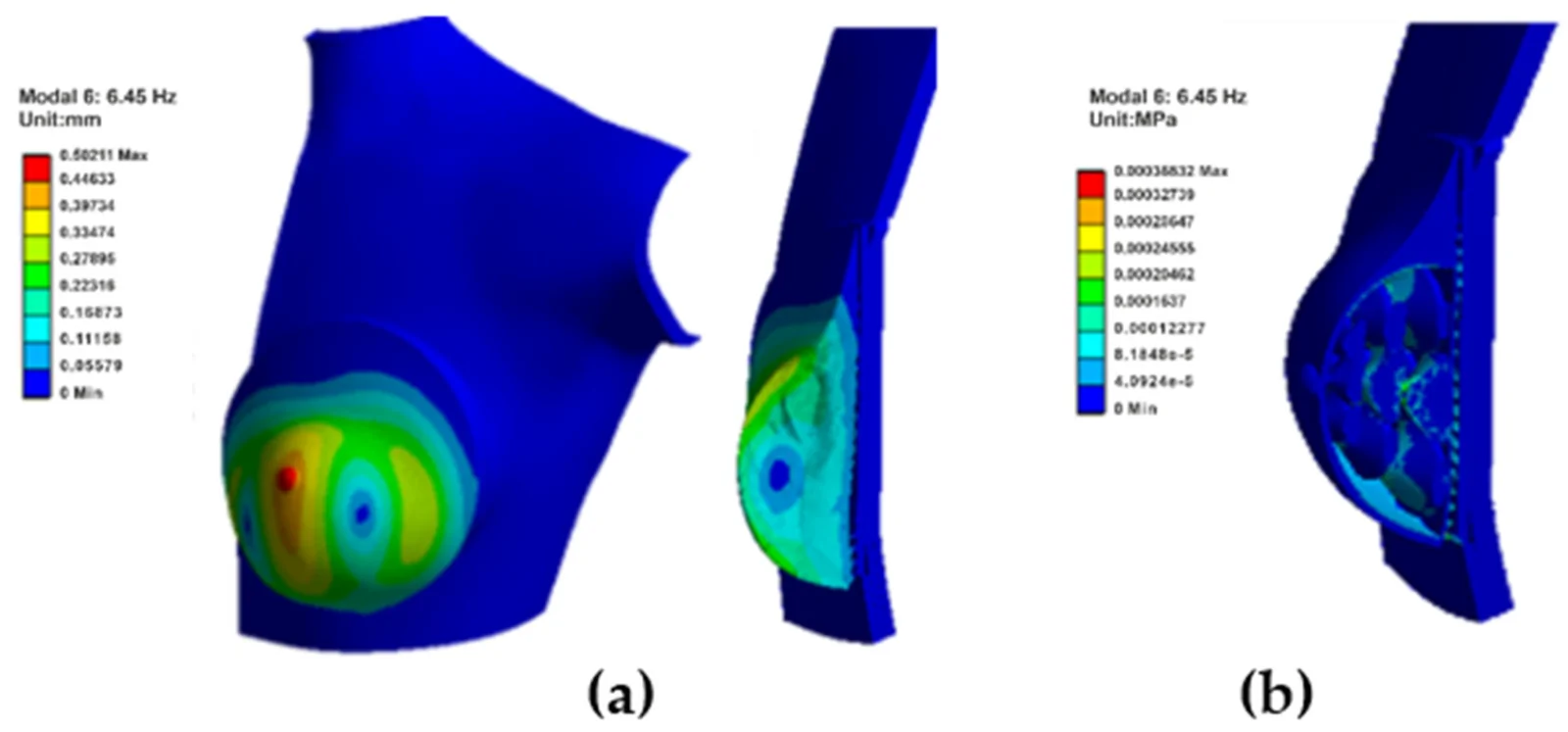
Sixth vibration mode of the prosthesis–torso assembly (6.45 Hz). (**a**) Total displacement (max: 0.49 mm). (**b**) Equivalent stress distribution.

The sixth vibration mode (Figure 10a) occurred at a frequency of 6.45 Hz and corresponded to a combined linear–torsional left-to-right motion, similar to the previous mode, with a maximum amplitude of 0.49 mm. Internal deformations were uniformly distributed, and stresses were primarily concentrated within the internal lobular structure (Figure 10b), thereby avoiding critical stress concentrations and without producing significant alterations in the torso.

Table 1 compares the natural frequencies obtained in this study with those reported by Cruz et al. (2018) [6] and Chen L. H. et al. (2013) [5,16,22]. For the first four vibration modes, reasonable agreement was observed in both modal order and frequency ranges, with relative differences attributable to variations in geometry, material properties, boundary conditions, and modeling assumptions across the respective numerical simulations. Modes 1 and 2 fall within the 3–4 Hz interval, while higher-order modes were concentrated between 4 and 6 Hz, consistent with values reported for biomechanical systems of similar characteristics. The first four modes showed a similar modal order and comparable frequency intervals, with relative differences of approximately 2.5–16.3% compared with the results reported by Chen et al. [16].

Furthermore, previously unidentified modes (5–8) were identified in the range of 6.3–7.2 Hz, expanding the dynamic characterization of the prosthesis–torso system under excitations associated with daily activities. The resulting natural frequencies lie outside the dominant physiological excitation range associated with gait (≈1–3 Hz).

The results of the modal analysis indicated an ergonomically acceptable dynamic behavior, as the system response lies within the physiological range reported for soft breast tissue under normal dynamic conditions [6]. No critical deformations or stress concentrations appeared within the prosthesis operating range. The internal lobular structure effectively fulfilled its support and energy dissipation functions, contributing to the overall system stability. The prosthesis–torso system exhibited stable and safe dynamic performance under expected use conditions. The agreement between the natural frequencies obtained here and those reported in the literature supports the biomechanical plausibility of the proposed biomimetic design approach and its feasibility for external breast prosthesis applications.

Overall, the modal results provide a comparative characterization of the natural frequencies and normalized deformation patterns of the proposed prosthesis–torso model. Their general agreement with previously reported numerical frequency intervals supports the consistency of computational formulation.

### 3.1. Harmonic Analysis

Harmonic analysis was employed to evaluate the vibratory response of the prosthesis–torso system under periodic excitation near resonance conditions. A harmonic force of 5 N was applied to the upper external surface of the torso, representative of typical human manual contact forces (1–20 N) [25]. Under this excitation, a frequency sweep was performed over the same range considered in the modal analysis over the same 3–9 Hz interval.

**Figure 11:**
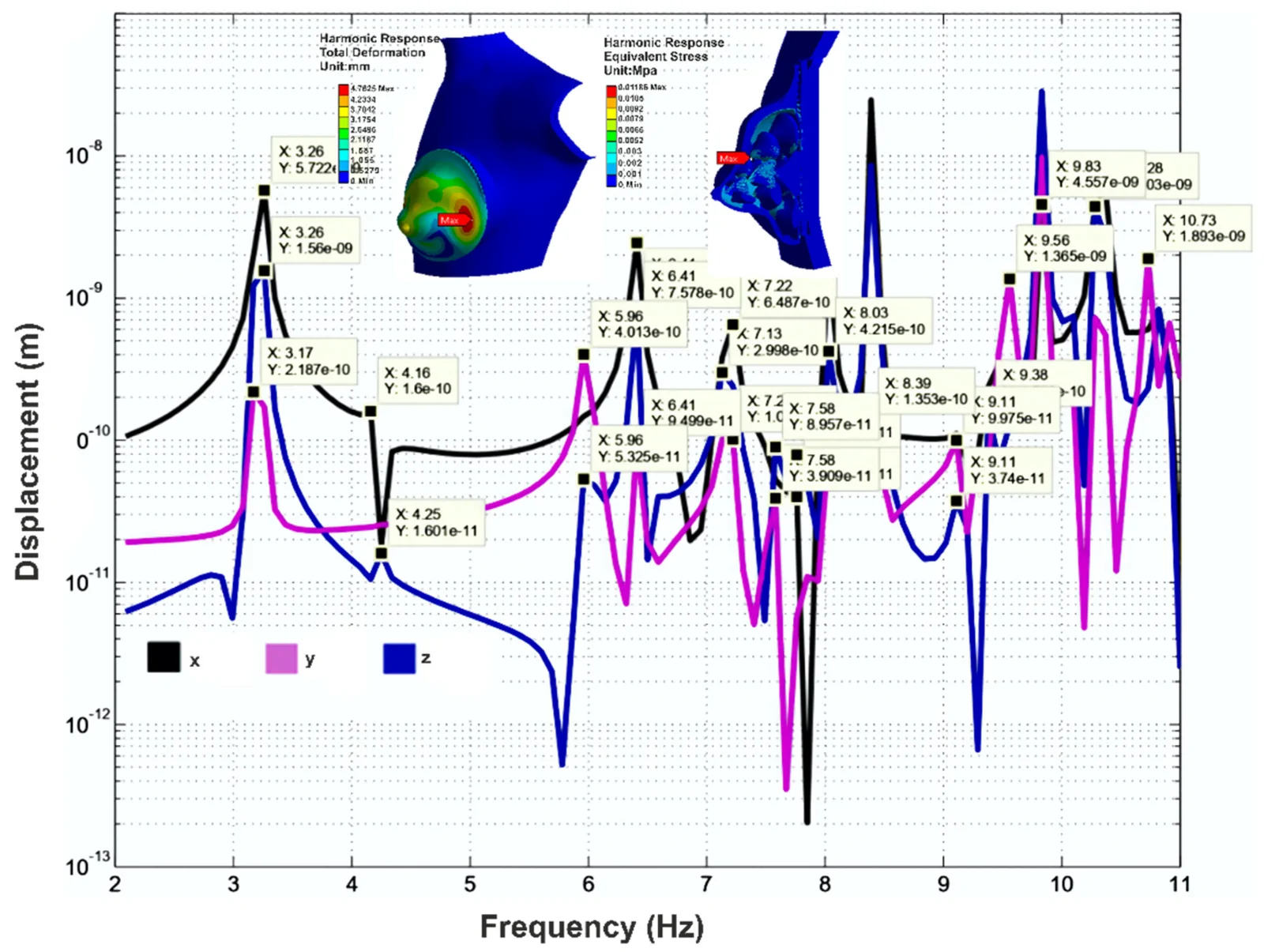
Harmonic response of the prosthesis–torso system. Frequency–displacement curves showing resonance peaks within 3–9 Hz and corresponding deformation and stress distributions.

The model exhibited a maximum deformation of approximately 4.7 mm in the region of highest displacement, as shown in the total-deformation inset in Figure 11. The equivalent- stress inset shows that the predicted internal stress levels remained below 1 MPa. The frequency-response curves exhibited displacement peaks corresponding to the resonance frequencies of the evaluated configurations.

The frequency response exhibited displacement peaks corresponding to the vibration modes identified in the modal analysis, primarily in the nipple region for Modes 1, 6, and 8. Despite the resonance-related amplification, the resulting deformations remained within physiologically acceptable ranges for soft tissue motion under representative daily-use conditions. The largest displacements were concentrated mainly in the nipple region.

Viscous material behavior and structural damping were not included in the harmonic model. The harmonic analysis indicates that, under the modeled 5 N periodic excitation and continuous-contact condition, the internal stresses remained below the reported numerical threshold. Altogether, these results support the dynamic stability of the prosthesis–torso system and its capacity to absorb harmonic loads without generating critical stress concentrations, thereby contributing to ergonomic performance and user comfort during normal operating conditions.

### 3.2. Spectral Analysis

**Figure 12:**
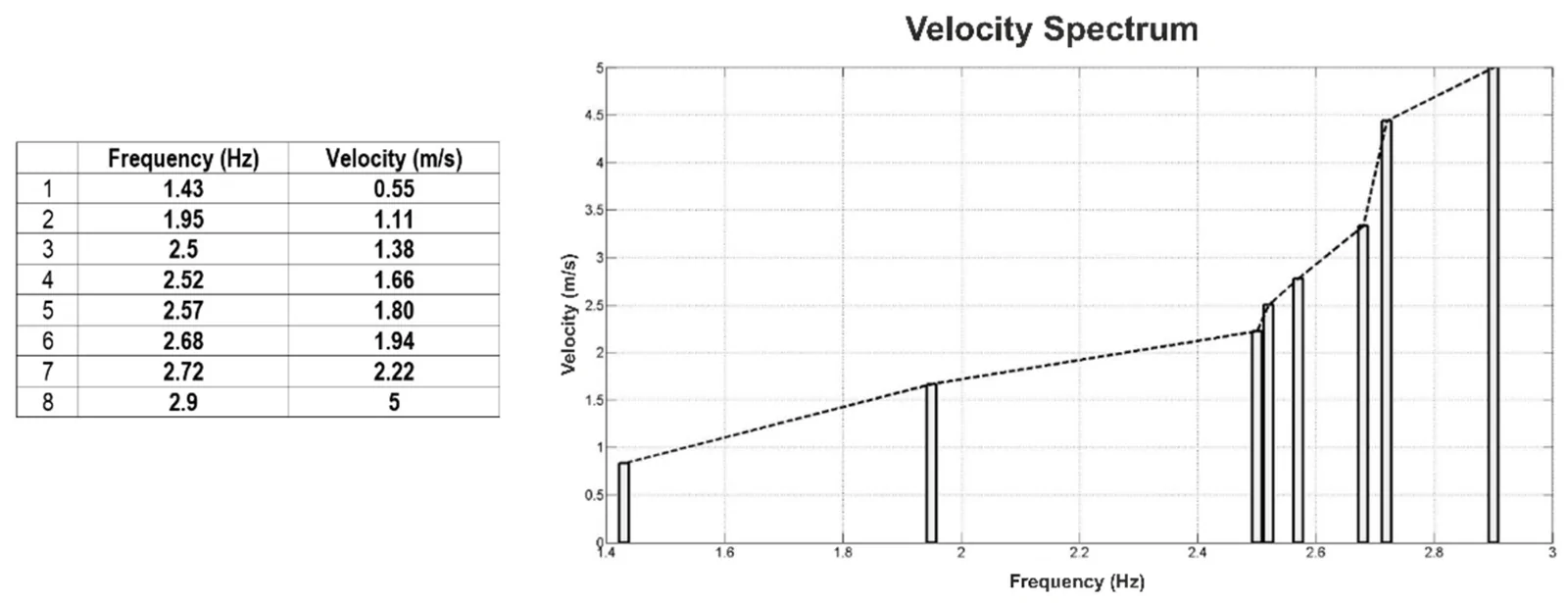
Frequency–velocity spectrum of human gait used as spectral input for the prosthesis dynamic analysis.

From a biomechanical perspective, spectral analysis of the prosthesis was conducted to characterize its natural frequencies, vibration modes, and dynamic displacements under loads associated with daily activities and to compare its response with the dynamic behavior of natural tissues and joints. This study focused on evaluating the performance of a breast prosthesis during gait, considering the dynamic spectrum of a person walking with a gradual increase in speed. Figure 12 shows the frequency–velocity relationship used to estimate the dynamic impact on the prosthesis under these conditions [23]. The excitation represented a gradual increase in walking velocity and was used to estimate its effect on the dynamic displacement of the prosthesis.

**Figure 13:**
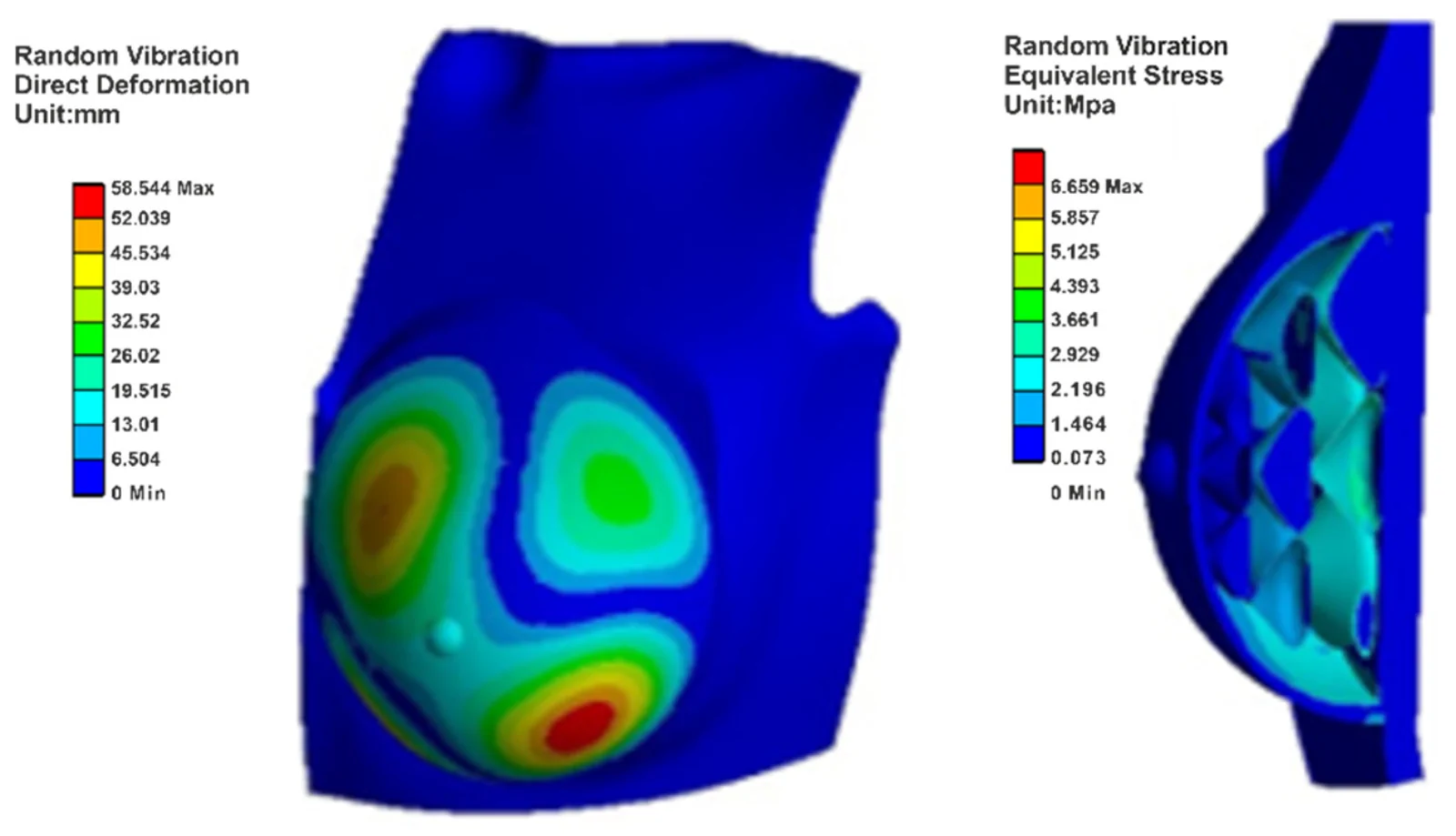
Total deformation and equivalent stress distribution of the prosthesis–torso model during walking conditions.

A spectral load was applied to the torso of the model to evaluate the dynamic displacements of the prosthesis during walking. The maximum predicted deformation under this walking spectrum attained approximately 50 mm (Figure 13), consistent with the behavior reported for real breast tissue during daily activities (40–70 mm) [26,27]. The stress distribution indicated that the internal structure of the model absorbed the applied loads, preventing critical stress concentrations.

**Figure 14:**
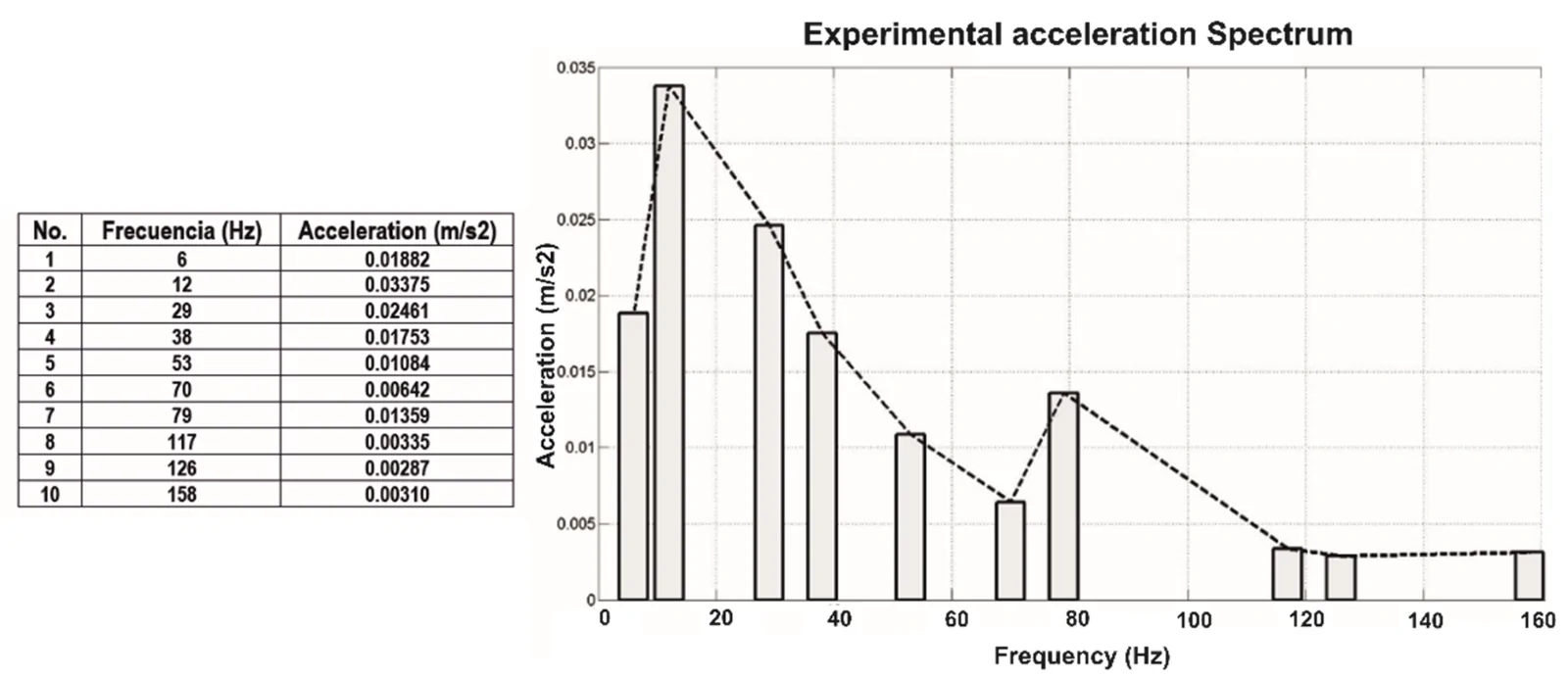
Experimental acceleration spectrum (frequency vs. acceleration) representing vibration conditions during public transportation. Authors’ reconstruction based on data reported in Ref. [24], with aceleration converted from g to m/s^2^.

Likewise, the prosthesis–torso model was subjected to a vibration spectrum representative of daily activities, such as public transportation use, considering a frequency range from 0.5 to 80 Hz, which was reported as ergonomically acceptable for preserving user health [28]. The characteristic frequency and acceleration data for this type of excitation are shown in Figure 14. The loading condition was analogous to that in the previous case, with excitations applied to the torso to evaluate their influence on the dynamic response of the prosthesis.

**Figure 15:**
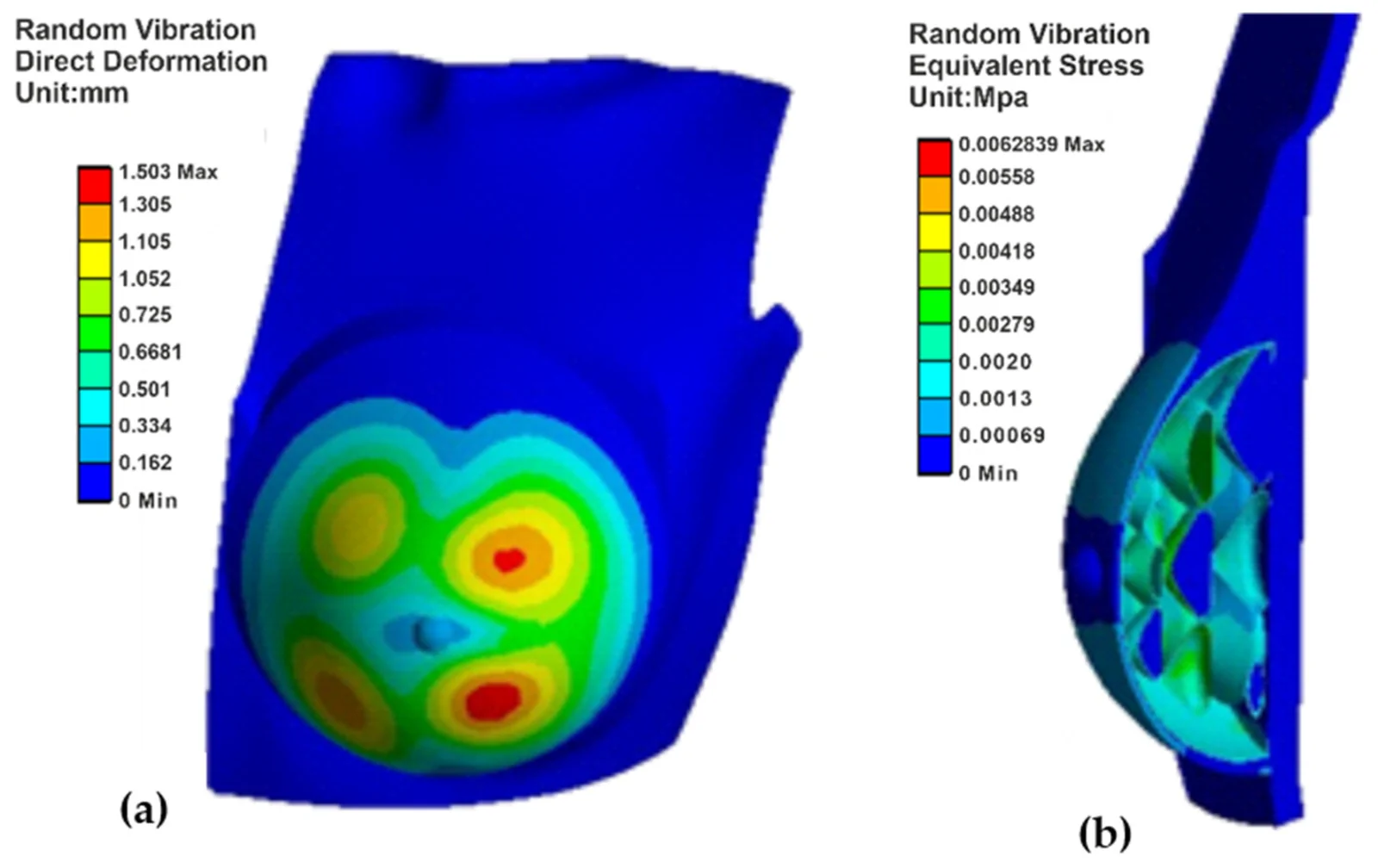
(**a**) Total deformation and (**b**) equivalent stress distribution of the prosthesis–torso model under public transportation vibration conditions.

Figure 15a shows that the deformation generated during public transportation use was minimal (≈1.5 mm) and practically imperceptible. As in the previous case, stresses were concentrated and absorbed by the internal structure of the model, preventing their propagation toward the periphery, as shown in Figure 15b, where the internal lobules play a key role in this process.

The different analyses must be interpreted separately. The modal analysis produced normalized mode shapes rather than physical displacement amplitudes. The harmonic analysis predicted a maximum displacement of approximately 4.7 mm under a controlled 5 N undamped excitation. The walking-related spectral case produced a maximum predicted deformation of approximately 50 mm, whereas the transportation-related spectrum produced approximately 1.5 mm.

Collectively, the spectral simulations show that the predicted response depends strongly on the excitation spectrum applied to the torso. The numerical stress patterns suggest that the internal lobular geometry contributes to distributing the modeled loads throughout the prosthesis. The spectral analysis demonstrated that, under vibrations associated with daily activities such as walking, running, or public transportation use, the prosthesis exhibited deformations within the range of 1–5 mm, consistent with physiologically acceptable soft tissue motion reported in the literature. Together with the displacement amplitudes obtained from the modal (<0.5 mm) and harmonic (~4.7 mm) analyses, these results indicate adequate load absorption and distribution by the internal biomimetic structure without compromising mechanical stability [29].

### 3.3. Thermal Analysis

**Figure 16:**
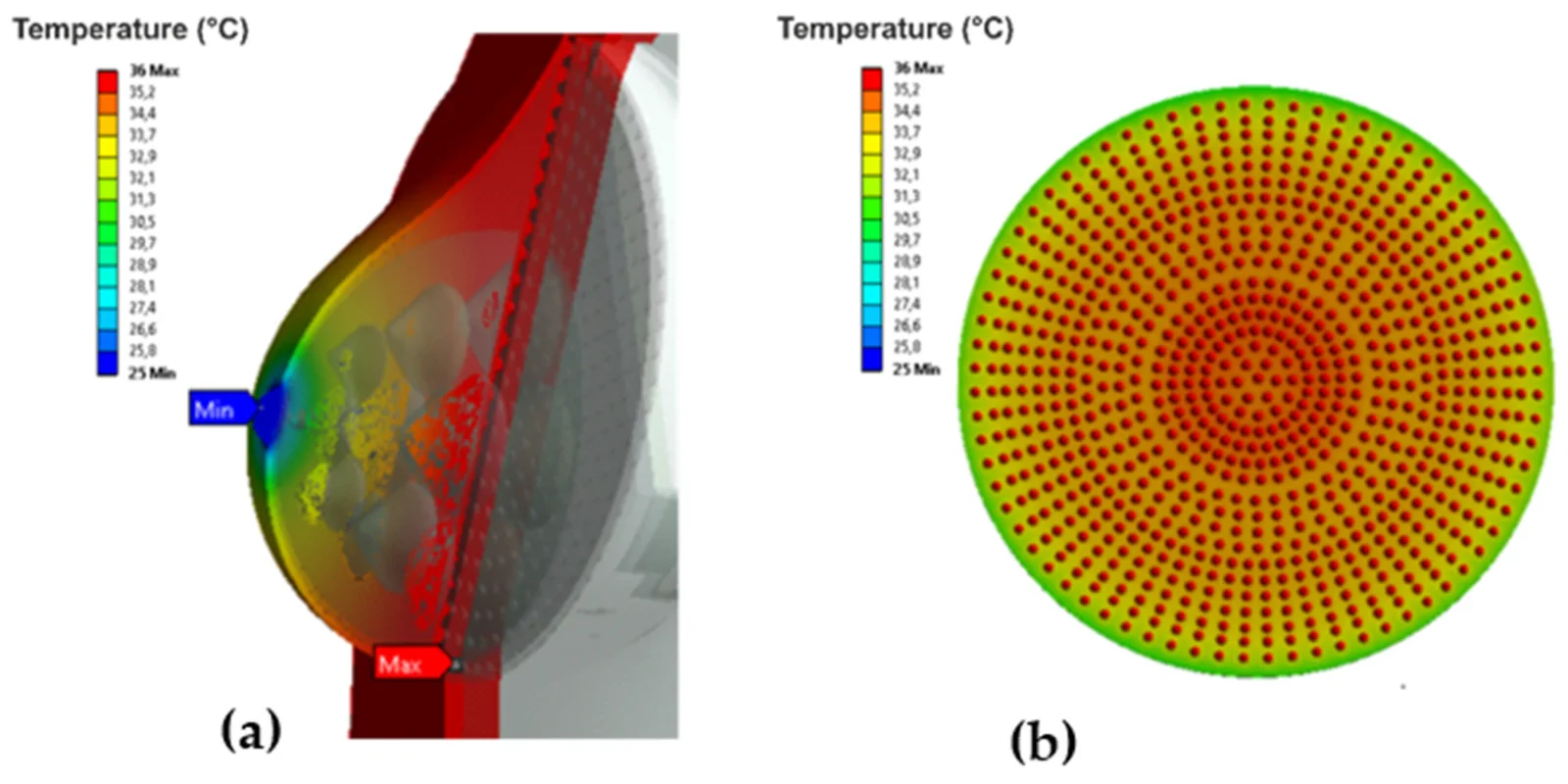
Steady-state temperature distribution of the prosthesis–torso assembly under controlled thermal conditions. (**a**) Longitudinal section (**b**) Posterior contact surface.

A steady-state thermal analysis was performed under controlled prosthesis–torso interaction conditions. The model assumed continuous contact associated with an adhesive layer and garment support. The torso-interface temperature was set to 36.00 °C [17]. Radiation, blood perfusion, metabolic heat generation, perspiration, evaporation, and physiological thermoregulation were not included, and the ambient temperature was considered at 25 °C (Figure 16a). Results showed uniform heat transfer, with temperature approximating 35.2 °C in the contact area and 25 °C at the opposite end, without heat concentration. Consistently, the adhesion region exhibited homogeneous thermal dispersion throughout the geometry (Figure 16b).

The steady-state thermal analysis demonstrated uniform heat distribution throughout the prosthesis–torso interface without critical thermal concentrations. Nevertheless, the resulting contact temperature (~35.2 °C) remained above the target interface temperature range established in the evaluation criteria, emphasizing the need for improved ventilation mechanisms to enhance heat dissipation and user comfort.

### 3.4. Ventilation Analysis

**Figure 17:**
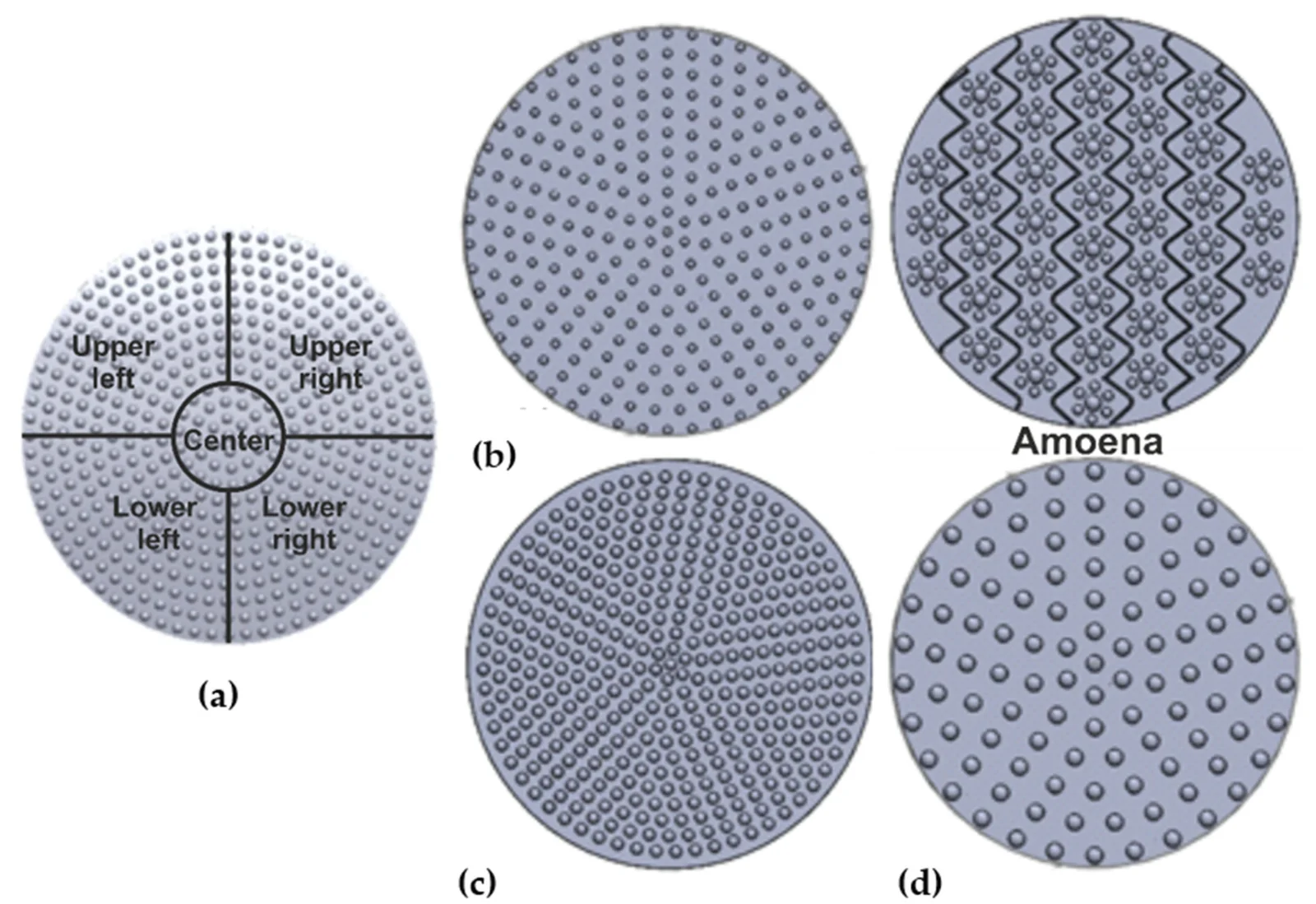
Posterior surface configurations evaluated for ventilation analysis: (**a**) reference model with defined quadrants, (**b**–**d**) sphere arrangement patterns, and commercial model with protrusions.

The ventilation analysis evaluated airflow and sensible heat transfer at the posterior prosthesis–torso interface under simulated dry-air conditions. Ventilation plays a key role in thermal and user comfort during prosthesis use [29]. In this study, ventilation analysis was employed to evaluate airflow behavior at the prosthesis–torso interface and its influence on thermal distribution in the proposed models. The model was divided into five reference point sections (Figure 17), assuming an average air temperature of 24.5 °C, an air velocity of 0.25 m/s, and a convective heat-transfer coefficient of 5 W·m^−2^·K^−1^. Corresponding to indoor comfort conditions established by NOM-001-STPS-2008. A relative humidity of approximately 40% was considered only as contextual environmental information. Humidity was not incorporated as a transported variable, and the model did not include perspiration, surface wetting, evaporation, condensation, sweat generation or moisture transport.

Three sphere arrangement configurations on the posterior surface of the prosthesis were evaluated (Figure 17) and compared with a commercial model incorporating protrusions designed to promote ventilation and reduce sweating in the torso contact area (Figure 17).

**Figure 18:**
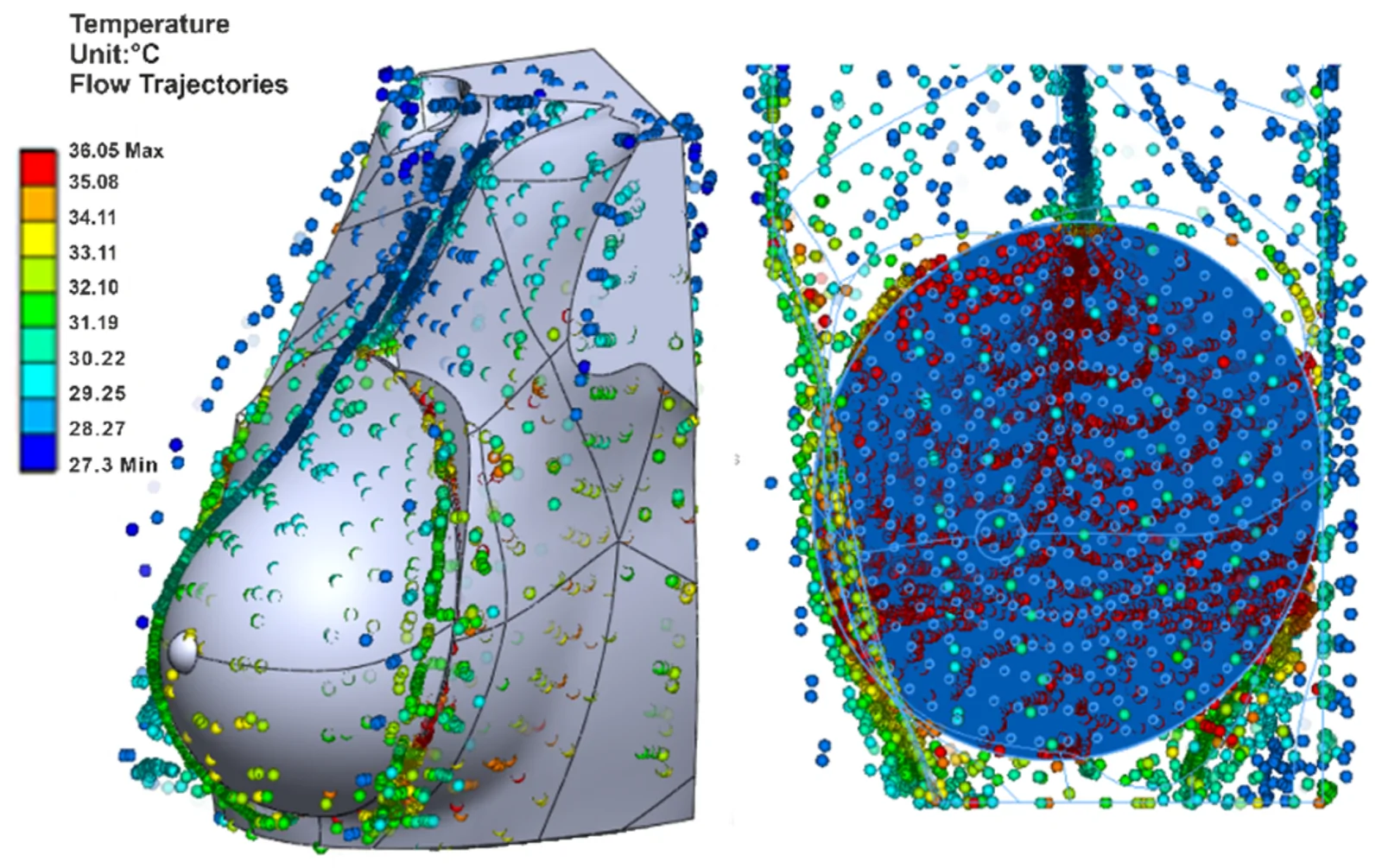
Airflow distribution and temperature field at the prosthesis–torso interface under controlled ventilation conditions.

All configurations were analyzed using the same material properties, thermal and airflow boundary conditions, contact assumptions, and numerical settings. Figure 18 shows that simulated airflow patterns, when interacting with the body of the wearer, are redirected toward the prosthesis–torso interface, promoting adequate heat dissipation. Likewise, the proposed protrusions proved effective, as air is distributed through the spheres under the established environmental conditions, contributing to a reduction in model temperature.

**Figure 19:**
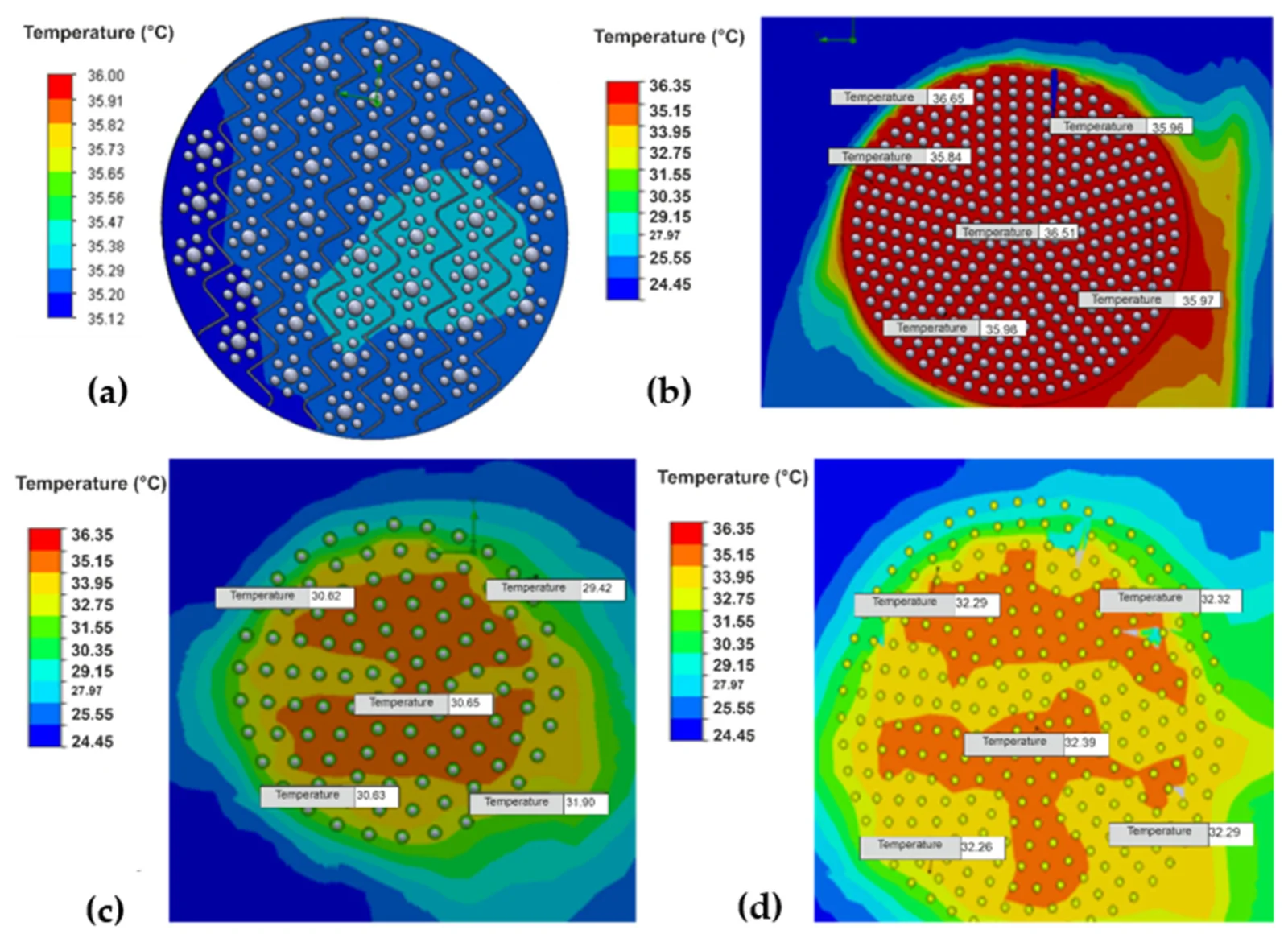
Temperature distribution on the posterior surface for the four evaluated configurations: (**a**) commercial model (Amoena) and (**b**–**d**) proposed sphere-based designs.

The four configurations shown in Figure 19 were evaluated under identical conditions to analyze fit and thermal distribution on the posterior prosthesis surface. The commercial Amoena-based configuration exhibited limited cooling capacity (35.38–35.12 °C), indicating that its protrusion pattern does not effectively contribute to temperature reduction in the contact area. Proposal 1 (Figure 19) showed a uniform thermal distribution but no cooling effect. In contrast, Approach 2 produced the greatest predicted temperature reduction. Its average interface temperature was 30.52 °C, corresponding to an average decrease of 5.48 °C relative to the prescribed initial interface temperature of 36.00 °C. The minimum localized temperature was 29.42 °C, representing a maximum localized numerical difference of 6.58 °C. Compared with the average temperature of the commercial configuration, Approach 2 produced an average interface temperature approximately 4.73 °C lower.

Approach 3 produced an average interface temperature of 32.41 °C, corresponding to an average reduction of 3.59 °C relative to the initial 36.00 °C condition and a temperature approximately 2.84 °C lower than the average commercial-model value.

Overall, the proposed sphere-based configurations achieved greater temperature reductions than the reference commercial model under the evaluated conditions, indicating improved airflow circulation and heat dissipation at the prosthesis–torso interface.

A summary of the evaluation of the four models is presented in Table 2.

A quantitative comparison of the four evaluated configurations revealed consistent temperature differences across all measurement regions. The average interface temperatures were 35.91 °C for Approach 1, 30.52 °C for Approach 2, 32.41 °C for Approach 3, and 35.25 °C for the commercial Amoena prosthesis. Although Approach 2 achieved the largest temperature reduction, decreasing the average interface temperature by approximately 4.73 °C compared with the commercial reference, Approach 3 provided a temperature reduction of 2.84 °C while maintaining improved anatomical fit and reduced visibility under clothing. Since all numerical models were evaluated using identical boundary conditions, material properties, and mesh settings, the observed temperature differences can be attributed primarily to geometric modifications rather than numerical discretization effects. These results support the selection of Approach 3 as the most balanced design from both thermal and ergonomic perspectives.

## 4. Discussion

The ergonomic evaluation of the prosthesis for complete mastectomy cases enabled assessment of its dynamic, mechanical, and thermal behavior under representative idealized loading and environmental conditions. In the absence of specific regulatory standards for external breast prostheses, we interpreted results based on quantitative criteria derived from the literature on soft tissue biomechanics, human body vibration, and cutaneous heat dissipation. Nevertheless, the reported displacement, stress, temperature, and airflow values are computational predictions and should not be interpreted as experimentally validated measures of in-use performance or human comfort.

From a dynamic perspective, the modal analysis covered the 3–9 Hz interval and identified eight natural frequencies within this range. The first four natural frequencies differed by approximately 2.5–16.3% from the values reported by Chen et al. [16]. Differences observed relative to previous studies can be attributed to variations in prosthesis geometry, the incorporation of an internal lobular structure, and contact conditions defined with the torso. Low-frequency modes associated with global displacements were identified outside the dominant range of physiological excitation during human gait (≈1–3 Hz), thereby reducing the probability of resonance during daily activities. Higher-order modes exhibited uniform deformations without localized stress concentrations that could compromise structural integrity. The low-frequency location of several natural modes indicates that their possible interaction with gait-related or other periodic excitations should be examined experimentally. A reduction in resonance risk during daily activities cannot be established from the present analysis because complete subject-specific gait conditions were not reproduced and viscous damping was not included.

The harmonic analysis was performed using a controlled periodic force of 5 N. Gravity was defined as 9.81 m/s^2^, and the prosthesis–torso interface was represented using an idealized continuous-contact condition associated with the combined retention provided by medical adhesive and garment support. Under these numerical conditions, maximum displacements were on the order of 4.7 mm and stress levels well below the elastic limit of the material. Although these amplitudes exceed the fine mechanical perception threshold (<1 mm), they lie within the range of normal physiological displacements of breast tissue during daily activities [20,21], indicating a biomechanically compatible response consistent with the soft and deformable nature of the tissue. The frequency response exhibited peaks coincident with the modes identified in the modal analysis, without excessive amplification or unstable behavior, reflecting adequate capacity for energy absorption and dissipation attributable to the internal lobular structure. These results should be interpreted as comparative numerical results rather than validated in-use amplitudes. Viscous material behavior and structural damping were not incorporated into the harmonic analysis. Consequently, the predicted amplitudes, particularly those close to the natural frequencies, may be greater than the amplitudes occurring during actual use. The results therefore demonstrate long-term, relative frequency-dependent response of the modeled configuration but do not establish perceived comfort or dynamic stability in a user.

Spectral analysis under excitations representative of gait confirmed global deformations on the order of several millimeters, consistent with the dynamic behavior of a natural breast during locomotion [29]. Under vibrations associated with public transportation, deformations were considerably smaller (≈1–5 mm) and practically imperceptible, demonstrating the ability of the design to adapt to different dynamic scenarios without compromising stability or generating discomfort.

With respect to structural behavior, stress generated in all analyzed scenarios remained well below the critical limits of the material, with no localized concentrations or regions susceptible to damage. Homogeneous stress distribution reinforces the functional role of the internal structure in mechanical stability and device durability.

Furthermore, a direct mechanical comparison between the biomimetic lobular architecture and a conventional homogeneous prosthesis was not performed. The comparison with the Amoena-type commercial pattern was limited to posterior thermal and airflow behavior under identical simulated conditions.

Regarding thermal behavior, all posterior configurations were evaluated using the same dry-air numerical conditions; sweat may partially obstruct the spaces between the microspheres and increase airflow resistance. Evaporation may also contribute to heat removal; however, moisture transport was not included in the present CFD model.

The initial torso-interface temperature was set to 36.00 °C [17], the inlet air temperature to 24.5 °C, the inlet velocity to 0.25 m/s, and the convective heat-transfer coefficient to 5 W·m^−2^·K^−1^. A relative humidity of approximately 40% was considered only as contextual environmental information and was not included as a transported variable in the computational model. The average interface temperatures obtained for the evaluated configurations were 35.91 °C (Approach 1), 30.52 °C (Approach 2), and 32.41 °C (Approach 3), compared with 35.25 °C for the Amoena type reference configuration. Relative to the initial interface condition of 36.00 °C, Approach 2 produced an average predicted temperature reduction of 5.48 °C and a maximum localized reduction of 6.58 °C, corresponding to a local temperature decrease from 36.00 °C to 29.42 °C. Approach 3 produced an average predicted reduction of 3.59 °C. Relative to the average temperature predicted for the Amoena-type configuration, the average temperatures of Approaches 2 and 3 were lower by approximately 4.73 °C and 2.84 °C, respectively. The comparison baseline must therefore be stated explicitly when reporting these differences.

These numerical reductions are comparable to the temperature decreases reported in recent studies investigating ventilated prosthetic interfaces and biomimetic soft-tissue devices, where enhanced airflow pathways and porous structures contributed to improved heat dissipation. Unlike conventional solid prostheses, the sphere-based posterior architecture promoted air circulation within the prosthesis–torso interface, reducing thermal accumulation under identical environmental conditions. However, it should be noted that the present study was based exclusively on numerical simulations; therefore, the observed temperature reductions indicate improved thermal dissipation potential rather than a demonstrated increase in user comfort.

The selection of Approach 3 reflects a multicriteria compromise between thermal performance, anatomical fit, and aesthetic discretion. Although Approach 2 achieved the lowest interface temperature, the smaller sphere diameter adopted in Approach 3 reduced the prosthesis–torso separation distance and minimized potential visibility under clothing while still maintaining an average temperature reduction of approximately 8.1% relative to the commercial reference.

### 4.1. Limitations

Although the study is based on an idealized numerical model and does not account for phenomena such as actual sweating, active muscle activity, or interindividual variability among users, the results provide a solid basis for the ergonomic evaluation of external breast prostheses.

In addition, the constitutive formulation assumed isotropic and nearly incompressible hyperelastic behavior without incorporating viscoelasticity or anisotropic tissue responses, which may influence long-term dynamic and thermomechanical behavior under repetitive loading conditions. The absence of viscoelastic material behavior and damping may affect the predicted dynamic amplitudes, particularly near the natural frequencies.

Taken as whole, the evidence presented suggests that the proposed design demonstrates improved thermal dissipation and biomechanical compatibility under the evaluated numerical conditions and a significant improvement in comfort, supporting its feasibility as a functional solution for everyday use.

### 4.2. Applicability

To enhance the scalability and reproducibility of the proposed biomimetic approach, a parametric CAD framework was developed through an application programming interface (API) integrated into a SolidWorks^®^ environment.

**Figure 20:**
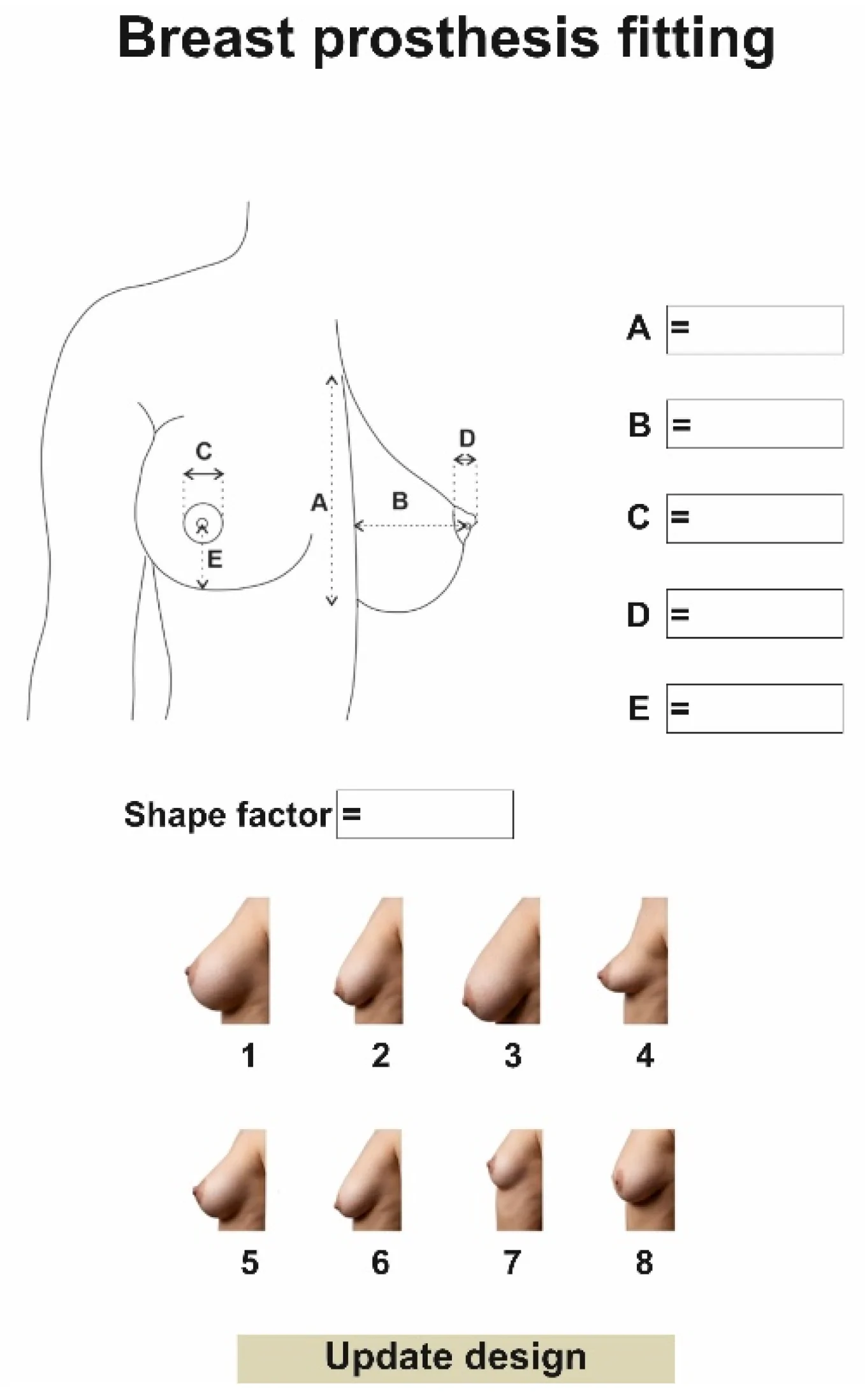
Graphical user interface of the SolidWorks^®^ macro developed in Visual Basic for parametric breast prosthesis fitting and automatic model updating.

The developed framework enables systematic updating of prosthesis geometry based on patient-specific anthropometric parameters, facilitating reproducible customization and automated geometric adaptation (Figure 20).

Through the interaction window, patient-specific anthropometric parameters are entered, along with a shape factor associated with the silhouette that best matches the morphology of the healthy breast. In cases of bilateral mastectomy, the system automatically selects an optimal geometry based on the user’s body build.

Once the anthropometric parameters are defined, the framework automatically updates the external geometry and internal biomimetic structure while preserving the predefined design relationships and functional characteristics of the prosthesis. The resulting fully parameterized three-dimensional model is suitable for numerical evaluation and future fabrication processes. The parametric strategy also enables preservation of internal biomimetic architecture during geometric scaling and customization, maintaining the functional principles associated with load distribution and thermal dissipation.

This integration establishes a reproducible CAD–CAE workflow that facilitates future incorporation into digital manufacturing and multi-material additive manufacturing processes for personalized prosthesis fabrication.

Manufacturing feasibility, dimensional accuracy, material durability, and clinical suitability remain to be demonstrated experimentally.

### 4.3. External Validation of the Parametric Model and Derived Contributions

The proposed biomimetic and parametric approach has subsequently been adopted and extended in related studies focused on advanced prosthesis design and additive manufacturing applications. In particular, the study by Reinoso Hayashi (2023) [30], conducted at Delft University of Technology, adopted the internal architecture inspired by the lobular arrangement of breast tissue proposed by Cruz et al. (2018) [6], highlighting the conceptual robustness and transferability of the model to advanced design and manufacturing contexts.

That study demonstrated that a parametric geometric model can be effectively integrated with high-resolution multi-material additive manufacturing technologies, such as voxel-based PolyJet printing, while maintaining geometric coherence and control over internal mechanical properties without requiring complete structural redesigns. These findings reinforce the scalability and adaptability of the proposed parametric approach across different manufacturing contexts.

Rather than constituting an independent experimental validation, these subsequent developments demonstrate the scalability and adaptability of the proposed parametric and biomimetic framework across different manufacturing and design scenarios. However, results obtained with a different geometry, material system, manufacturing process, and testing protocol cannot be used as direct validation of the current numerical model.

In terms of mechanical and perceptual responses, the reported results suggest that the combination of a biomimetic internal structure, controlled material distribution, and a flexible outer shell may achieve a consistency comparable to that of natural breast tissue. Mechanical testing and tactile evaluations performed in the referenced study indicated that material consistency plays an important role in the perception of both physical and psychological comfort, providing indirect evidence consistent with the functional rationale of the proposed biomimetic structure. Nevertheless, they provide only indirect contextual support and do not establish that the present design reproduces the consistency of natural breast tissue or improves physical or psychological comfort.

The study also pointed out that, although the use of statistical models enables high aesthetic fidelity of external geometry, customization of the chest wall contacts surface remains an area requiring further development. This observation reinforces the relevance of the parametric CAD framework proposed in the present work, as it facilitates the incorporation of patient-specific anthropometric parameters and advances toward automated personalized prosthesis design.

Finally, the identified limitations related to flexible material durability, geometric adaptability at reduced scales, and the printability of large multi-material volumes should not be interpreted as weaknesses of the proposed framework, but rather as directions for future research. Overall, these findings suggest that the proposed biomimetic and parametric methodology constitutes a scalable and reproducible foundation for the future development of personalized external breast prostheses.

## 5. Conclusions

In this work, an external breast prosthesis for complete mastectomy cases was developed using a parametric CAD framework and evaluated through mechanical, dynamic, thermal, and airflow simulations. The analyses were conducted under representative idealized loading, contact, and dry-air environmental conditions and therefore characterize the numerical behavior of the proposed design rather than its actual performance during clinical or everyday use.

The numerical analyses showed dynamically stable behavior, with natural frequencies located outside the dominant range of physiological excitation and displacement amplitudes within the range reported for soft breast tissue motion during daily activities. Furthermore, the internal lobular architecture was associated with a more homogeneous load distribution and improved structural stability under the evaluated mechanical conditions.

The thermal and airflow simulations showed that the posterior microsphere configurations generated convective airflow pathways and lower predicted interface temperatures under the simulated dry-air conditions; sweat and evaporation were not considered. The selected configuration, Approach 3, produced an average interface temperature of 32.41 °C, compared with 35.25 °C for the Amoena-type reference configuration, representing a numerical difference of 2.84 °C under identical boundary conditions. Although Approach 2 produced a greater temperature reduction, Approach 3 was selected as a design compromise because its smaller microspheres reduced the prosthesis–torso separation and the potential visibility of the posterior texture beneath clothing. These results indicate improved sensible heat dissipation but should not be interpreted as evidence of enhanced human heat dissipation perception, since sweating, evaporation, physiological thermoregulation, clothing effects, and user perception were not modeled.

The proposed parametric CAD framework enabled automated updating of prosthesis geometry from anthropometric parameters, facilitating reproducible customization and providing a basis for future integration into CAD–CAE–additive manufacturing workflows for personalized prosthesis fabrication. Anthropometric relationships were developed from a convenience sample of 30 participants, whereas the numerical case evaluated in this study was generated from measurements of one anatomical medical mannequin. Therefore, population-level applicability, subject-specific fit, and clinical suitability have not yet been established.

The findings of this numerical study suggest that biomimetic geometric features may contribute to improved mechanical and thermal performance compared with conventional solid configurations. However, because the analyses were based on computational models and did not include experimental or clinical validation, the results identify potentially useful mechanical and thermal characteristics under the evaluated numerical conditions, but they do not demonstrate clinical effectiveness, improved user comfort, mechanical superiority, or long-term durability.

Future work should include the manufacture of multi-material prototypes and controlled mechanical experimental validation under compression, cyclic loading, and harmonic excitation. Displacement, interface pressure, natural frequencies, damping, stress relaxation, and permanent deformation should be measured and compared with numerical predictions. Thermal and airflow validation should employ a temperature-controlled torso phantom or thermal manikin, infrared thermography, embedded temperature sensors, and dry- and wet-condition testing. Silicone deformation and sweat accumulation may modify the dimensions and connectivity of the posterior airflow channels, affecting airflow resistance, contact area, and heat transfer. Subsequent evaluations with representative users will be required to assess fitness, aesthetic acceptability, perceived comfort, durability, and the clinical relevance of the proposed methodology.

## Figures and Tables

**Table 1:** Comparison of the natural frequencies obtained with those reported.

Mode	Frequency Obtained (Hz)	Frequency (Hz) [6]	Frequency (Hz) [16]
1	3.1849	3.59	4.87
2	3.2419	3.64	4.91
3	4.2307	5.05	4.99
4	5.9884	5.84	5.02
5	6.3922	-	-
6	6.4571	-	-
7	7.1802	-	-
8	7.1962	-	-

**Table 2:** Steady-state temperature comparison (°C) for the evaluated posterior surface configurations.

Region	Temperatures (°C)			
	Approach 1	Approach 2	Approach 3	Amoena
Upper left	35.74	30.62	32.28	35.4
Upper right	35.81	29.42	32.32	35.23
Center	36.36	30.65	32.37	35.29
Lower left	35.83	30.63	32.72	35.2
Lower right	35.82	31.29	32.37	35.12

## Data Availability

Available upon reasonable request.

## Abbreviations

The following abbreviations are used in this manuscript:

CAD	Computer-Aided Design
CAM	Computer-Aided Manufacturing
CAE	Computer-Aided Engineering
FEM	Finite Element Method
CFD	Computational Fluid Dynamics
CMM	Coordinate Measuring Machine
API	Application Programming Interface
